# The tumour suppressor CCDC6 is involved in ROS tolerance and neoplastic transformation by evading ferroptosis

**DOI:** 10.1016/j.heliyon.2021.e08399

**Published:** 2021-11-15

**Authors:** Francesco Morra, Francesco Merolla, Federica Zito Marino, Rosaria Catalano, Renato Franco, Paolo Chieffi, Angela Celetti

**Affiliations:** a Institute for the Experimental Endocrinology and Oncology, Research National Council, CNR, Naples, Italy; b Department of Medicine and Health Sciences “V. Tiberio”, University of Molise, Campobasso, Italy; c Pathology Unit, University of Campania “L. Vanvitelli”, Naples, Italy; d Department of Psychology, University of Campania “L. Vanvitelli”, Naples, Italy

**Keywords:** Seminoma, Embryonal carcinoma, DNA DSBs, Homologous recombination, PARP inhibitors, Deferoxamine, Ferrostatin-1, xCT/SLC7A11 cystine antiporter, Erastin, Targeted therapies, Glutathione

## Abstract

Coiled-coil domain containing 6 (CCDC6) is a tumour suppressor gene involved in apoptosis and DNA damage response. CCDC6 is known to be functionally impaired upon gene fusions, somatic mutations, and altered protein turnover in several tumours. Testicular germ cell tumours are among the most common malignancies in young males. Despite the high cure rate, achieved through chemotherapy and/or surgery, drug resistance can still occur. In a human cellular model of testis Embryonal Carcinoma, the deficiency of CCDC6 was associated with defects in DNA repair via homologous recombination and sensitivity to PARP1/2 inhibitors. Same data were obtained in a panel of murine testicular cell lines, including Sertoli, Spermatogonia and Spermatocytes. In these cells, upon oxidative damage exposure, the absence of CCDC6 conferred tolerance to reactive oxygen species affecting regulated cell death pathways by apoptosis and ferroptosis. At molecular level, the loss of CCDC6 was associated with an enhancement of the xCT/SLC7A11 cystine antiporter expression which, by promoting the accumulation of ROS, interfered with the activation of ferroptosis pathway. In conclusion, our data suggest that the CCDC6 downregulation could aid the testis germ cells to be part of a pro-survival pathway that helps to evade the toxic effects of endogenous oxidants contributing to testicular neoplastic growth. Novel therapeutic options will be discussed.

## 1 Introduction

Testicular germ cell tumours (TGCTs) represent one of the most common malignancies in young men [1]. TGCTs are divided into pure seminoma and nonseminomatous germ cell tumours [2], which include embryonal cell carcinoma, choriocarcinoma, yolk sac tumour and teratoma.

In the differentiation process, the death or survival of the different populations of the testis depends on the balance between survival and regulated cell death signals in response to reactive oxygen species. Among the regulated cell death programmes, ferroptosis has been recently characterized as an iron- and lipid reactive oxygen species (ROS)-dependent form of death, distinct from other patterns of regulatory cell death at the morphological, biological, and genetic levels [3], [4]. Emerging evidence suggests critical roles for ferroptosis in various diseases, including cancers [5]. Moreover, ferroptosis can be inhibited in different cancer types and may function as a dynamic tumour suppressor in cancer development, also suggesting that the regulation of ferroptosis can be utilized as an interventional target for tumour treatment [6].

The molecular mechanisms at basis of the TGCTs are poorly understood [7]. In these tumours, the rate of reported somatic mutations is low, despite the high levels of aneuploidy. Expression of the KIT tyrosine kinase is critical for normal germ cell development and is observed in the majority of seminoma [8]. In these tumours the KIT oncogene is the most frequently mutated gene, with a rate of mutations ranging from 18% to 31%, and exon 17 is the most affected region, mainly on D816 residue [9]. Mutations in codon 12, 13, and 61 of KRAS and NRAS, which cause a decrease in GTPase activity, with a consequent constitutive activation of the encoded proteins, have been also described, albeit with a lower frequency [10], [11], [12].

Interestingly, the TGCTs represent the most chemosensitive solid tumours [13]. However, the mechanisms behind their sensitivity, mostly related to an insufficient DNA repair of cisplatin-induced DNA adducts, and to a hypersensitive apoptotic response, remain elusive. The cytotoxicity of cisplatin can be ascribed to defective double strand break (DSB) repair by homologous recombination (HR). An impairment of DNA DSBs repair by HR could also determine sensitivity to Poly(ADP-ribose) polymerase (PARP) inhibitors. Indeed, a high sensitivity to PARP inhibitors, alone or in combination with cisplatin, have been reported in a panel of TGCTs cell lines, letting envisage an additional therapeutic approach to utilize in tumours testis [14]. Nevertheless, there is an urgent need to identify specific and reliable biomarkers able to predict the PARP-inhibitors sensitivity.

In response to DNA damage, the recruitment of the Serine/Threonine (S/T) ATM kinase at sites of DNA breaks leads to the activation of the (S/T) ATM kinase signaling with phosphorylation of nearly 700 substrates, which include MDM2, p53, and also the Coiled Coil Domain Containing 6 (CCDC6) gene products [15]. The tumour suppressor CCDC6 is a ubiquitously expressed nuclear and cytosolic protein, phosphorylated at Serine 244 residue by ERK1/2 and able to induce apoptosis. CCDC6 is known to be functionally lost upon gene translocations, somatic mutations, and altered protein levels in several tumours [16]. Additionally, the *in vitro* conditional expression of CCDC6 truncated mutants, which act as dominant negative of the endogenous wild type protein, determines tolerance to oxidative damage in cancer cells, impairing the stress-induced cell death [15], [16], [17]. In response to genotoxic stress CCDC6, phosphorylated by the (S/T) ATM kinase at the residue Thr434, translocates to the nucleus where it participates to the homologous recombination machinery through the histone H2AX phosphorylation by negatively regulating the PP4c phosphatase activity [18]. The impairment of ERK1/2-mediated Serine 244 phosphorylation and of ATM-mediated Threonine 434 phosphorylation on CCDC6 protein, result in tolerance to oxydative and genotoxic stress, affecting the CCDC6 proapoptotic activity and its involvement in DNA Damage Response (DDR). Most importantly, preclinical studies indicate that the attenuation of CCDC6 in lung, pleural, prostate and bladder cancer determines cells sensitivity to inhibitors of PARP1/2 enzymes [19], [20], [21], [22].

In this work our intent has been to investigate the expression levels of CCDC6 in several cellular models of murine testis, where CCDC6 showed different level of expression. Notably, the CCDC6 deficiency, which associates with an impairment of the HR DNA DSBs repair, determined PARP inhibitor sensitivity in testicular cancer models. Moreover, the CCDC6 depletion conferred tolerance to oxidative damage, resulting associated with enhanced expression and activity of the xCT/SLC7A11 cystine antiporter [23], [24], leading to evasion of regulated cell death pathways of apoptosis and ferroptosis.

In the present investigation we tested the hypothesis that the CCDC6 loss may contribute to the testicular germ cell transformation process. In the future, a better understanding of the mechanisms which associate CCDC6 with ferroptosis could provide references for novel potential targets to use in the treatment of testicular cancers.

## 2 Materials and methods

### 2.1 Cell lines, drugs and chemicals

Human Embryonal Carcinoma cell line NTERA-2 and the murine testicular cell lines TM4 (Sertoli), GC-1 (Spermatogonia), and GC-2 (Spermatocytes) were maintained in DMEM (Gibco, Paisley, UK), supplemented with 10% fetal bovine serum (FBS; Gibco BRL, Italia), 1% L- Glutamine and 1% of penicillin – streptomycin (Gibco, Paisley, UK) [25], [26], [27], [28]. Olaparib (AZD2281) and P005091 were provided by SelleckChem. Cycloheximide, cisplatinum, H2O2 (H1009), erastin (E7781), ferrostatin-1 (SML0583) and desferoxamine (D9533) were from Sigma-Aldrich, Inc (St. Louis, CA, USA). Z-VAD-fmk (FMK001) was from MedChemExpress.

### 2.2 Reagents and antibodies

For the biochemical analysis the following antibodies were utilized: anti-CCDC6 (ab56353) Abcam, (Cambridge, UK), anti-tubulin (T6557) Sigma-Aldrich, anti-PCNA (NANO3), anti-USP7 (A300-033A) Bethyl, Inc (Montgomery, TX, USA), anti-xCT/SLC7A11 (D2M7A) Cell Signaling, Inc (Danvers, MA, USA) and anti-Myc clone 9E10 (sc-40) Santa Cruz Biotechonology, Inc (Dallas, TX, USA). For the immunoistochemical analysis the antibodies anti-CCDC6 (HPA 019051), from Sigma-Aldrich, and the anti-xCT/SLC7A11 (D2M7A), from Cell Signaling, were utilized. The secondary antibodies were from Biorad (Hercules, CA, USA).

### 2.3 Sensitivity test and design for drug combination

Antiproliferative activity was determined by the CellTiter 96 AQueous One Solution Cell Proliferation Assay (Promega), in terms of 50% inhibitory concentration (IC50) values. The cells were plated in triplicate in 96-well plates at a density of 1000 cells per well, and continuously exposed to each drug for 144h. Each assay was performed in triplicate and IC50 values were expressed as mean +/- standard deviation.

### 2.4 Cell viability assay

The cells were plated in triplicate in 96-well plates at a density of 1000 cells per well and treated for 18h with H2O2 or Erastin in presence or not of Z-VAD-fmk, ferrostatin-1, deferoxamine. Cell viability was determined by the CellTiter 96 AQueous One Solution.

### 2.5 Protein extract and western blot analysis

Total cell extracts (TCE) were prepared with lysis buffer (50 mM Tris-HCl pH 7.5, 150 mM NaCl, 1% Triton X-100, 0.5% Na Deoxycholate, 0.1% SDS) and a mix of protease inhibitors. Protein concentration was estimated by a modified Bradford assay (Biorad). For Western blotting, cell lysates were separated by SDS-PAGE (10% polyacrylamide) and the proteins were transferred to a PVDF membrane. Membranes were blocked with 5% TBS-BSA and incubated with the primary antibodies. Immunoblotting experiments were carried out according to standard procedures and visualized using the ECL chemiluminescence system (Amersham, Little Chalfont, UK/Pharmacia Biotech, Milano, Italy). As a control for equal loading of protein lysates, the blotted proteins were probed with antibody against anti tubulin protein.

### 2.6 Plasmids and transfection

pcDNA4ToA -ev (empty vector), -mycCCDC6wt (wildtype), and -mycCCDC6T434A (mutant) plasmids were transfected with the FuGene HD (Promega) reagent. The mutant pcDNA4ToA-mycCCDC6T434A was generated as previously described [15]. CCDC6 shRNA (pLKO.1 puro) was from Sigma-Aldrich, Inc. Human NTERA-2 embryonal carcinoma cells and murine GC-1 cells were transfected with a pool of nontargeting vector (ShCTRL) or with a plasmid pool (shCCDC6, NM_005436) or (shCcdc6, NM_001111121.1) by Nucleofection for 48 hours, to obtain transient human or murine CCDC6 silencing, respectively. The pDR-GFP reporter plasmid is based on a construct developed by M. Jasin [29] and contains two mutated GFP genes separated by a puromycin drug selection marker.

### 2.7 HR assay

The cells were plated in a 60 mm plate and transfected with the pDR-GFP reporter alone (as negative control) or together with the pCAGGS-I-SceI plasmid, by the FuGene HD (Promega) reagent. After 48 hours cells were collected and analyzed by FACS analysis with BD Accuri C6 Flow Cytometer (BD Bioscience, Canada).

### 2.8 Real-time PCR

PCR reactions were performed on RNA isolated from cell lines using RNeasy Mini Kit (Qiagen) and reverse-transcribed using MuLV RT (Invitrogen). qRT-PCR was performed with Syber Green (Biorad) using the following primers: CCDC6 forward ggagaaagaaacccttgctg and reverse: tcttcatcagtttgttgacctga; mouse SLC7A11, forward gattcatgtccacaagcacac and reverse agagcatcaccatcgtcaga; human SLC7A11 forward gcgtgggcatgtctctgac and reverse gctggtaatggaccaaagacttc.

### 2.9 Immunohistochemistry

The adult murine sections of normal testis (4 μm) were immunostained with the antibodies anti-CCDC6 (HPA 019051), from Sigma-Aldrich, or with anti-xCT/SLC7A11 (D2M7A) (Cell Signaling) [22], [30]. IHC-stained glass slides were digitalized at 40x using the Leica Aperio AT2 slide scanner (Leica Biosystems) [31]. WSI images in .svs file format were analysed with QuPath platform [32]. The study was performed according to the Declaration of Helsinki and in agreement with the Italian and European laws on retrospective analyses on routine archival FFPE-tissue.

### 2.10 Cell blocks

Cells were seeded in 100 mm plates and collected at confluence, resuspended in 10% formalin and fixed for 12 hours before processing. Cell block preparation was performed according to manufacturer's instructions (7401150 - Thermo Fisher Scientific, UK) Samples were centrifuged for 1 minute at 2.5 rpm and supernatant discarded. Reagent 2 was added to the cell pellet. The Cytoblock cassette, cytoclip and cytofunnel were assembled. Three drops of Reagent 1 was added into centre of well of Cytoblock cassette and spun (cytospin) at 1500 rpm for 5 minutes on low acceleration. The Cytoblock cassette was closed and processed in the standard tissue processor [33].

### 2.11 GSH assay

GSH levels were detected using a GSH-Glo Glutathione Assay (Promega) following the manufacturer's instructions. Briefly, 1,000 cells per well were seeded in a 96-well plate 1 day before analysis. The culture medium from the wells was carefully removed, and 100 μl of prepared 1 × GSH-Glo Reagent was added, followed by incubation at room temperature for 30 min. Next, 100 μl of reconstituted Luciferin Detection Reagent was added to each well. After 20 min, luminescence was measured using a Gen5 Microplate reader. A standard curve for GSH concentration was generated, and exact GSH concentrations in different cell lines were calculated based on a GSH standard curve according to the manufacturer's instructions.

### 2.12 Lipid peroxidation assay

Lipid peroxidation levels were measured by BODIPY 581/591 C11 dye (Invitrogen, D3861). Briefly, cells were incubated in a 60 mm dish containing 5 μM BODIPY. After incubation for 30 min at 37 °C, cells were washed with PBS and trypsinized, then subjected to flow cytometry analysis using an Accuri C6 flow cytometer.

## 3 Results

### 3.1 Human Embryonal Carcinoma (EC) NTERA-2 cells and mouse testicular cells express different amount of CCDC6 at mRNA and protein level

**Figure 1:**
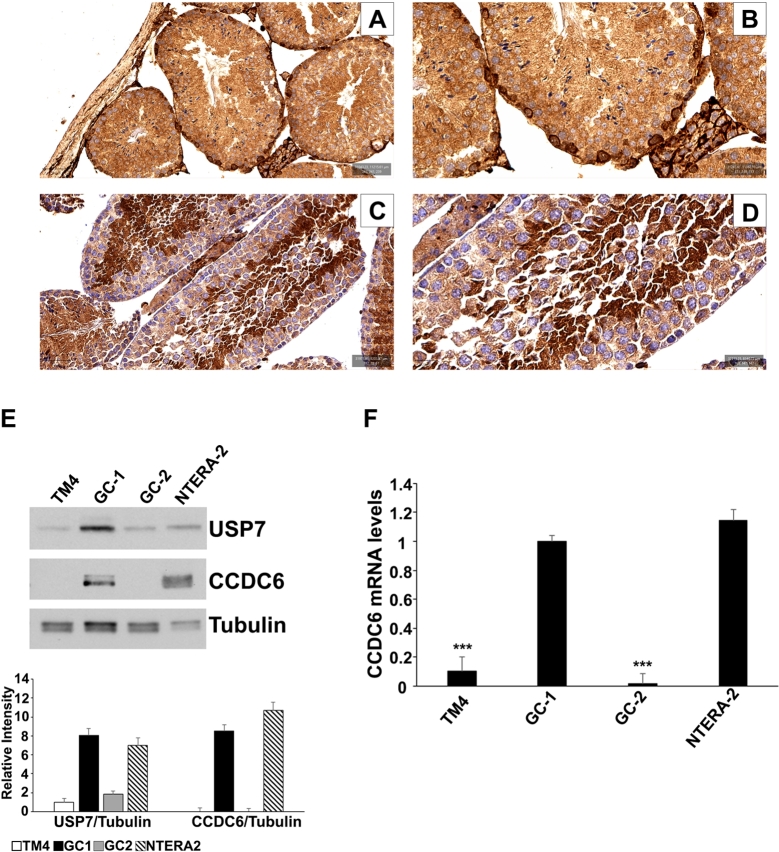
Immunohistochemical staining for CCDC6 and xCT/SLC7A11 proteins in adult mouse testis (DAB tecnique). A representative seminiferous tubule showing CCDC6 stronger expression in the basal germ cells (**A**, 20x; **B**, 40x) while xCT/SLC7A11 protein expression pattern is reverted (**C**, 20x; **D**, 40x). (**E**) Western Blot analysis of CCDC6 and USP7 expression levels in human NTERA-2 Embryonal Carcinoma cells, and in murine GC-1 Spermatogonia, GC-2 Spermatocytes and TM4 Sertoli cells. Tubulin is shown as loading control. (**F**) CCDC6 relative expression was assessed by RealTime PCR in the human NTERA-2 EC cells and in the murine GC-1, GC-2, and TM4 cells, as indicated. Statistical significance was verified by 2-tailed Student's t-test (* p <0.05; ** p <0.01 and *** p <0.001).

An immunohistochemical analysis to evaluate CCDC6 intensity of staining was performed on serial section of normal adult mouse testis. CCDC6 was observed in the germinal epithelium, particularly in the spermatogonia cells, which represent the basal compartment of the seminiferous tubule, anchored to the basal membrane. The CCDC6 detection was poorer in the spermatocytes and spermatids. The Sertoli cells, which are essential element of the seminiferous tubule as they structurally sustain the germ cells and supply them with nutrients, exhibited a barely appreciable amount of CCDC6, which is even less evident in the spermatozoa (Fig. 1 A, B). Then, by taking advantage of murine testicular cell lines corresponding to different types of testicular cell population, we evaluated the CCDC6 expression levels in protein extracts from *in vitro* Spermatogonia (GC-1), Spermatocytes (GC-2), and Sertoli (TM4) cells by western blot analysis (Fig. 1 E). The CCDC6 expression levels in testis cell lines tended to be consistent with the intensity of staining of the primary cells of the murine testis. Appreciable amount of CCDC6 was also detected at western blot in the NTERA-2 cells, a human cellular model of testis Embryonal Carcinoma (Fig. 1 E). Quantitative real time PCR analysis showed a good number of transcripts for CCDC6 in the NTERA-2 cells, as well as in GC-1 Spermatogonia cells. On the contrary, GC-2 spermatocytes and TM4 Sertoli cells showed low amount of transcripts for CCDC6 suggesting that the loss of CCDC6 protein in this cell types, might not be dependent on post-translational mechanism (Fig. 1 F).

### 3.2 Pharmacological inhibition of USP7 affects CCDC6 stability and impairs homology-directed repair (HDR) in Human Embryonal Carcinoma (EC) and murine testis cells

**Figure 2:**
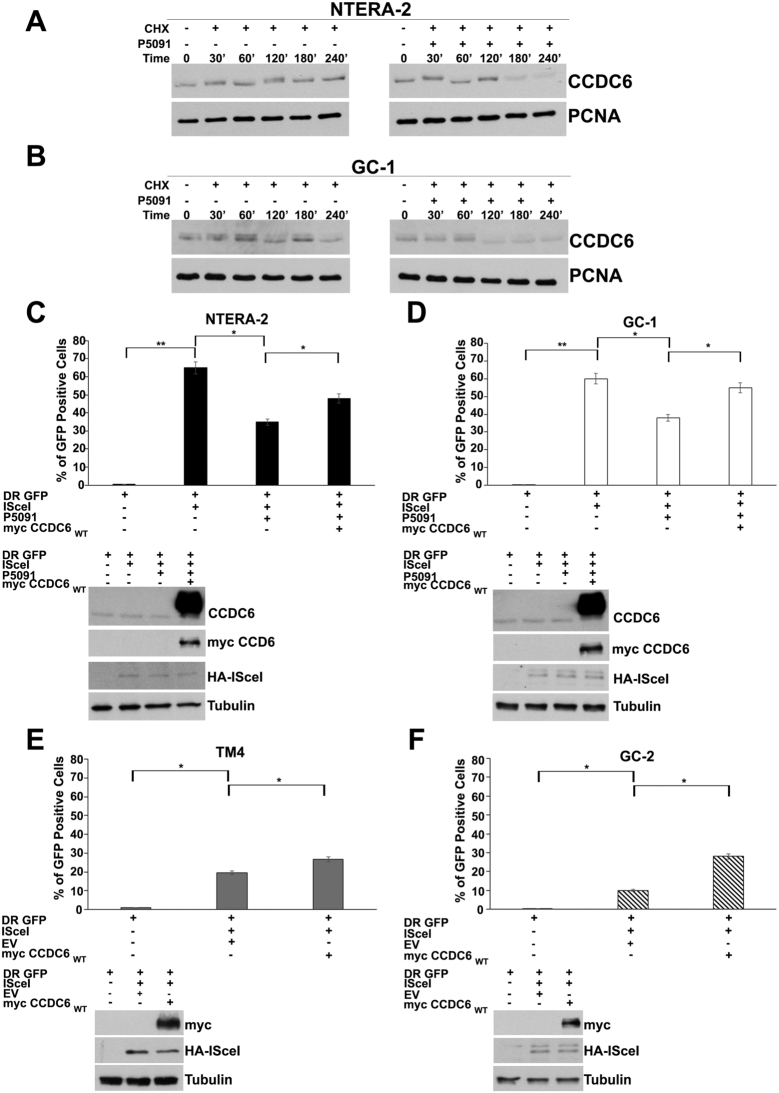
Pharmacological inhibition of USP7 affects CCDC6 stability and impairs homology-directed repair in Human Embryonal Carcinoma (EC) and murine cells of the testis. (**A**) Human NTERA-2 EC cells and (**B**) murine GC-1 Spermatogonia cells were pre-treated with either vehicle or P5091 (12.5 μM) for 4 h, followed by the addition of cycloheximide (CHX) at 50 μg/ml for the indicated times. Total proteins lysates were subjected to immunoblot analysis using anti-CCDC6 or anti-PCNA antibodies, as loading control. (**C**) The Human NTERA-2 EC cells and (**D**) the murine GC-1 Spermatogonia cells, silenced for CCDC6 upon pre-treatment with either vehicle or P5091 (2.5 μM) for 4 h were transfected with DR-GFP alone, as control, or together with the I-SceI enzyme. The percentages of GFP positive cells, compared to controls, were plotted as histograms, representative of the mean of three independent experiments. Error bars indicate the measurement of the standard error mean. (**E**) The spontaneously CCDC6 null murine TM4 Sertoli cells and (**F**) GC-2 Spermatocytes were transiently transfected with DR-GFP alone, as control, or together with the I-SceI enzyme and the HR-reporter assay was performed. The percentages of GFP positive cells, compared to controls, were plotted as histograms representative of three independent experiments. Error bars indicate the standard error mean. (**C-F**) For the rescue experiments, the human and murine cells were transiently transfected with myc-CCDC6 wild type, and exogenous myc-CCDC6 was assessed at Western Blot by the anti-myc antibody. Endogenous and exogenous CCDC6 were also revealed at anti-CCDC6 immunoblot. Anti-tubulin immunoblots are shown as loading control. Statistical significance was verified by 2-tailed Student's t-test (* p <0.05; ** p <0.01 and *** p <0.001).

CCDC6 stability is dependent on the USP7 de-ubiquitinase activity, which if pharmacological inhibited, by P5091, increases the turnover of the CCDC6 gene product [34], [35]. Moreover, the P5091 preclinical use has been recently reported in lung, pleural, prostate and bladder cancer cells, also in combination with PARP inhibitors [22], [36], [37]. Appreciable levels of CCDC6 and USP7 proteins were detected in NTERA-2 human Embryonal Carcinoma and in the murine GC-1 spermatogonia cells, while nearly undetectable amount was observed in GC-2 spermatocytes and TM4 Sertoli cells (Fig. 1 E). Upon treatment with P5091, followed by the addiction of cycloheximide, the CCDC6 half-life was reduced, as revealed at the immunoblot anti-CCDC6 in NTERA-2 and GC-1 cells (Fig. 2 A, B). Low levels of CCDC6 have been reported associated with an impairment of HR mechanisms [38]. In order to assess the efficacy of HR repair, the murine testicular cells and the human NTERA-2 EC cells, left untreated or following pretreatment with P5091, depending on the CCDC6 expression levels, were transfected with the DR-GFP reporter plasmid alone, as a control, or together with the I-SceI plasmid, able to induce DSBs. The ability to repair the DSBs by HR was measured by flow cytometry and the frequency of HR was reported as a percentage of GFP positive cells. In the CCDC6 proficient NTERA-2 EC and GC-1 spermatogonia cells, the treatment with USP7 inhibitor P5091 determined a significant decrease of the GFP positive cells, compared to non-treated cells suggesting that the reduction of CCDC6 levels affected the DNA repair by HR (Fig. 2 C, D). An impairment to repair the DSBs by HR was also observed in the spontaneously null spermatocytes GC-2 and Sertoli TM4 cells, that was reverted upon the myc-CCDC6 transient expression in the GC-2 and TM4 (Fig. 2 E, F), as well as in the P5091-treated, NTERA-2 EC and GC-1 cells indicating a specific role of CCDC6 on these matters (Fig. 2 C-F).

### 3.3 Human Embryonal Carcinoma (EC) NTERA-2 cells exhibit differential sensitivities to PARP-inhibitors, depending on CCDC6 expression levels

**Figure 3:**
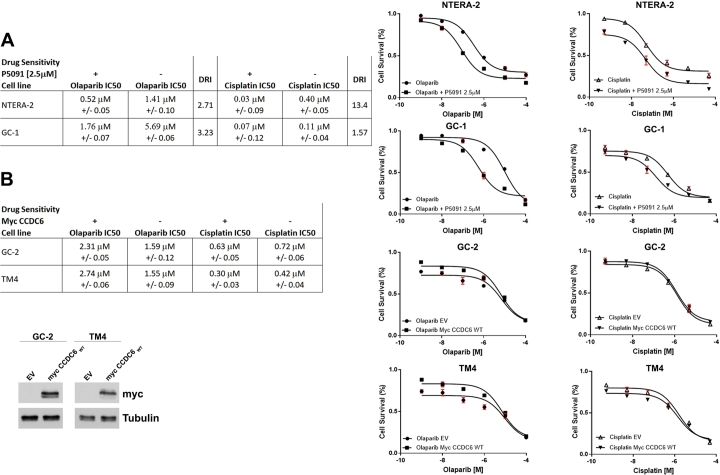
Human NTERA-2 Embryonal Carcinoma (EC) cells and murine cells of the testis exhibited differential sensitivities to PARP-inhibitors, depending on CCDC6 expression levels. Surviving fractions of (**A**) NTERA-2 and GC-1 cells and (**B**) GC-2 and TM4 cells treated with olaparib at the indicated doses for 144 h, in presence or absence of P5091 are shown. A Drugs sensitivity to olaparib and cisplatin, in presence or absence of P5091, was determined in human NTERA-2 EC cells and (**B**) in murine GC-1 spermatogonia cells by a modified 3-(4,5-dimethylthiazole-2-yl)-2-5-diphenyltetrazolium bromide assay, CellTiter 96 Aqueous One Solution assay (Promega), and expressed as IC50, i.e. the value that allows 50% of the inhibitory concentration. The Dose Reduction Index (DRI) for each drug reported in the table resulted >1, as the dose of olaparib to obtain the 50% Fractional Effect (IC50) resulted reduced upon the association of the P5091 at a fixed dose of 2.5 μM and of a range doses of olaparib, as indicated. Drugs sensitivity to olaparib and cisplatin was also determined, in the CCDC6 natively null GC-2 and TM4, in which myc CCDC6 or the EV were transiently re-expressed, by the CellTiter 96 Aqueous One Solution assay (Promega), and expressed as IC50, i.e. the value that allows 50% of the inhibitory concentration. CCDC6 was assessed at Western Blot by the anti-myc antibody. Anti-tubulin immunoblots are shown as loading control.

**Figure S1:**
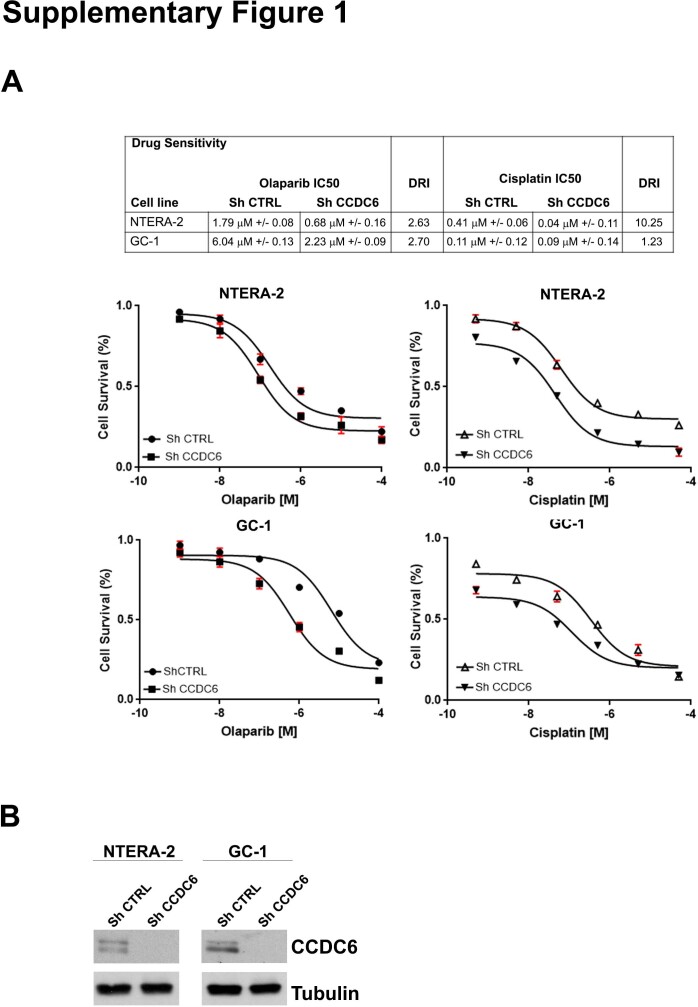
(**A**) Drugs sensitivity to olaparib and cisplatin, in human NTERA-2 EC cells and in murine GC-1 spermatogonia cells silenced or not for CCDC6, expressed as IC50. The Dose Reduction Index (DRI) for each drug reported in the table resulted >1, as the dose of olaparib to obtain the 50% Fractional Effect (IC50) resulted reduced upon the association of the CCDC6 silencing and of a range doses of olaparib, as indicated. (**B**) CCDC6 was assessed at Western Blot by the anti-CCDC6 antibody. Anti-tubulin immunoblots are shown as loading control.

Testicular germ cell tumours are chemosensitive with very high cure-rates even in the metastatic setting. However, patients resistant to platinum and with relapsing tumours have very poor outcome. No effective alternative therapies are available, highlighting the need for novel targeted therapies. Recently, the DDR pathway has been investigated as clinical target, since the TGCTs are characterized by defective repair of DNA DSBs and the targeting of the DDR components may provide a therapeutic opportunity. Indeed, the use of PARP1/2 ihibitors in cancer cells bearing DDR defects has been gaining always more attention [39], and *in vitro* data has shown that the treatment of TGCTs cells with PARP inhibitor olaparib are able to sensitize testicular cancer cells to cisplatin treatment, and in particular in case of cisplatinum resistance [14]. However, tumour biomarkers capable to predict the sensitivity to PARP inhibitors are highly envisaged but still missing. Since the HR defect is accompanied by sensitivity to PARP inhibitors, we aimed to characterise and quantify the outcomes of CCDC6 downregulation on olaparib sensitivity in cancer testicular cells by measuring cellular metabolic activity as an indicator of cell viability. To do so, we lowered CCDC6 protein levels by USP7 inhibitor P5091 and assessed sensitivity to a range of olaparib concentrations (Fig. 3 A, B). Similarly, cisplatin sensitivity was also assessed. High cytotoxic effects were achieved in P5091 pre-treated NTERA-2 cells when exposed to olaparib, whose sensitivity improved roughly of 3 times compared to controls (NTERA-2: IC50 1.41 μM -/+ 1.10 versus, in presence of P5091 at 2.5 μM, IC50 0.52 μM -/+ 0.05). Similarly, chemical downregulation of CCDC6 by P5091 leads to improved sensitivity to olaparib in spermatogonia GC-1 cells (IC50 5.69 μM -/+ 0.08 versus, in presence of P5091 at 2.5 μM, IC50 1.76 μM -/+ 0.07). Furthermore, sensitivity to cisplatin was also affected by P5091 in NTERA-2 cells (IC50 0.40 μM -/+ 0.05 versus 0.03 μM -/+ 0.09) and in GC-1 cells (IC50 0.11 μM -/+ 0.04 versus 0.07 μM -/+ 0.12). The results of the combined treatment seemed to be extremely relevant when we analyzed the Dose Reduction Index (DRI) that in clinical setting leads to reduced toxicity toward the host, while the therapeutic efficacy is retained [40]. Interestingly, in these cells, by combining P5091 and olaparib, we obtained a DRI >1, that suggested a great dose reduction, with reduced toxicity while retaining the therapeutic effect (Fig. 3 A). The chemical depletion of CCDC6 in combination with cisplatin treatment, also determined a DRI >1 in NTERA-2 and GC-1 cells (Fig. 3 A). Overlapping results were also obtained after silencing of CCDC6 with human shRNA in NTERA-2 cells and murine shRNA in GC-1 cells (Supplementary Figure 1).

Of note, the CCDC6 natively null GC-2 and TM4 cells exhibited olaparib sensitivity, which was impaired upon CCDC6 reconstitution following mycCCDC6 transient transfection (GC-2: IC50 1.59 μM -/+ 0.12 versus, in presence of myc-CCDC6, IC50 2.31 μM -/+ 0.05; TM4: IC50 1.55 μM -/+ 0.09 versus, in presence of myc-CCDC6, IC50 2.74 μM -/+ 0.06). These observations suggest that the response to the PARP inhibitor drugs specifically depended on the CCDC6 levels in the analysed testicular cells, as already reported in other cell systems [41], [42]. The cisplatin sensitivity was poorly modulated by the CCDC6 rescue in the murine testicular cells (Fig. 3 B).

### 3.4 The loss of function of CCDC6 induces tolerance to peroxide hydrogen-induced programmed cell death in human NTERA-2 embryonal carcinoma cells and in the murine testicular cells

**Figure 4:**
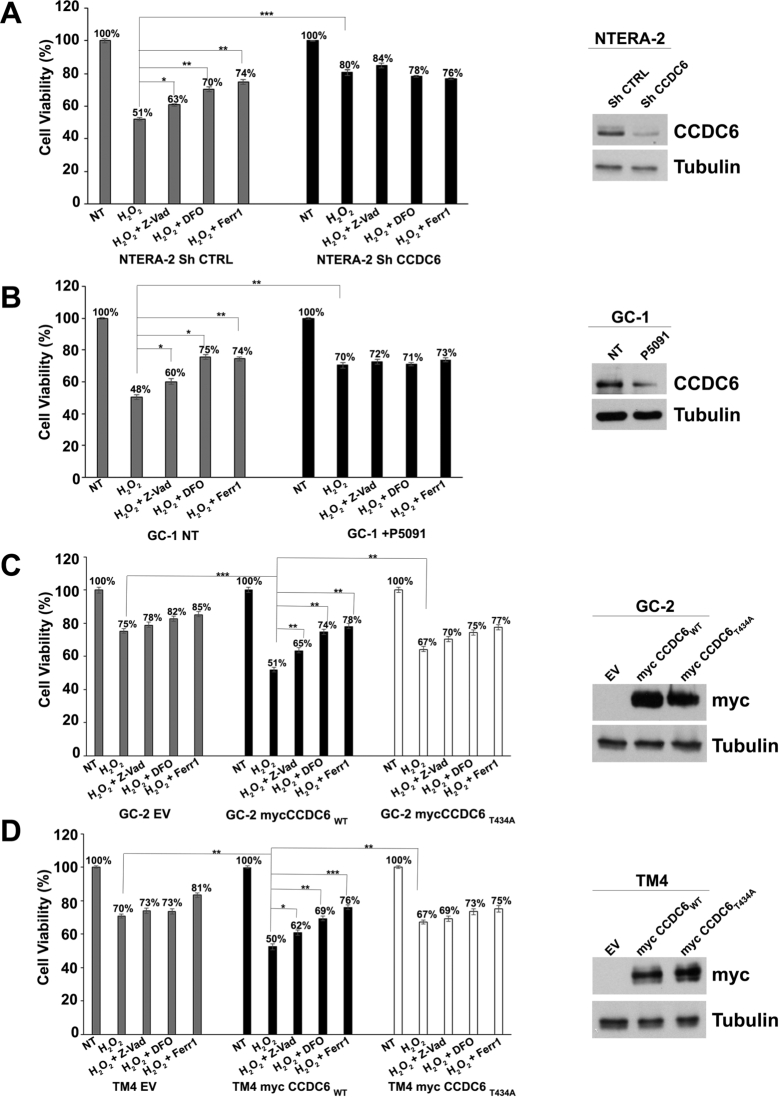
CCDC6 deficiency in human NTERA-2 cells and in the testicular murine cell lines induces ROS tolerance by impairing apoptosis and ferroptosis. Bar graphs showing cell viability of CCDC6-silenced (**A**) human NTERA-2 cells (upon transient transfection with shCCDC6 or control, shCTRL), (**B**) murine spermatogonia GC-1 cells (upon addition of P5091 or vehicle) and of CCDC6 natively null (**C**) murine spermatocytes GC-2 and (**D**) murine Sertoli TM4 cells (restored with empty vector (EV), as control, and with CCDC6wt or CCDC6T434A mutant), treated with ROS (600 μM H2O2) combined with 5 μM Z-VAD-fmk (Z-VAD), 2 μM ferrostatin-1 (Ferr-1) or 100 μM deferoxamine (DFO). CCDC6 was assessed at Western Blot by the anti-CCDC6 or anti-myc antibodies. Anti-tubulin immunoblots are shown as loading control. Error bars are mean ± s.d., of 3 independent repeats. Statistical significance was verified by 2-tailed Student's t-test (* p <0.05; ** p <0.01 and *** p <0.001). In **A**, **B**, **C**, and **D** percentage numbers have added to each histogram, as shown.

**Figure S2:**
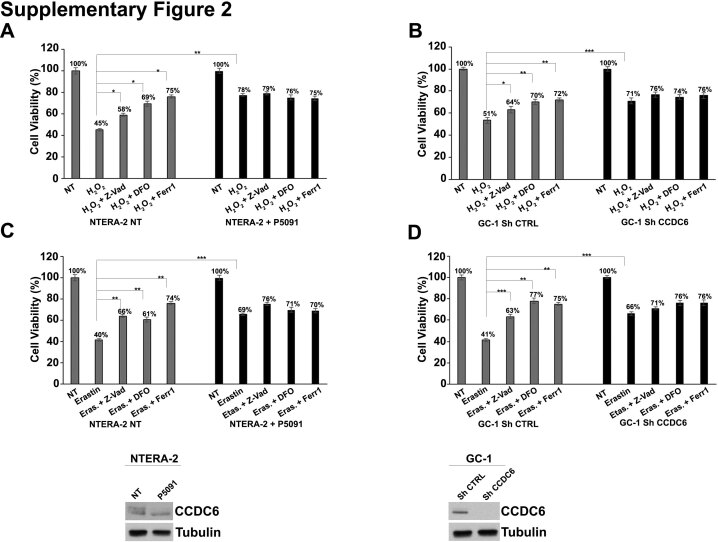
Bar graphs show cell viability, after treatment with H2O2 or Erastin, in (**A**, **C**) human NTERA-2 cells (upon addition of P5091 or vehicle), (**B**, **D**) in murine spermatogonia GC-1 cells (upon silencing of CCDC6 by transient transfection with shCcdc6 or control, shCTRL). In **A**, **B**, **C**, and **D** percentage numbers have added to each histogram, as shown.

In response to reactive oxygen species, an increasing number of regulated cell death (RCD) pathways have been characterized, including ferroptosis [43]. Moreover, a cross talk between apoptosis and ferroptosis has been recently reported [44]. In our previous work we observed that in human thyroid cells the overexpression of CCDC6 was capable to promote regulated cell death by apoptosis, while the overexpression of CCDC6 mutants or the CCDC6 depletion preserved cells viability, inducing tolerance to oxidative and genotoxic stress [15], [17]. In human NTERA-2 embryonal carcinoma cells, the CCDC6 depletion, upon a transient shCCDC6 transfection, prevented the cells death following exposure to hydrogen peroxide, in comparison to the control CCDC6 proficient cells (shCTRL) in which we observed a decrease of nearly 50% of the cells viability, limited by the apoptosis inhibitor Z-VAD-fmk (Fig. 4 A, left). Interestingly, in control cells, the cell death was also impeded by the iron chelator deferoxamine (DFO) and by the ferroptosis inhibitor Ferrostatin-1 [45]. DFO and Ferrostatin-1 (Ferr-1) did not affect viability in CCDC6 depleted, H2O2 treated cells (Fig. 4 A, right). The same results were obtained in the murine spermatogonia GC-1 cells upon CCDC6 chemical silencing by P5091, following hydrogen peroxide treatment, compared to vehicle treated cells (Fig. 4 B). Comparable results were observed upon P5091 chemical silencing of CCDC6 in NTERA-2 cells (Supplementary Figure 2 A) and following depletion of CCDC6 by murine shRNA in GC-1 cells (Supplementary Figures 2 B).

The viability of the Spermatocyte GC-2 and Sertoli TM4 cells, spontaneously null for CCDC6, were preserved following the peroxide hydrogen treatment (Fig. 4 C, D). However, in these cells, the transient overexpression of myc CCDC6wt, highly affected cells viability, mostly dependent on apoptosis and on ferroptosis pathways as reverted by Z-VAD-fmk, and by DFO and Ferr-1 pretreatment, respectively. The enforced expression of CCDC6 WT in H2O2-treated CCDC6 spontaneously null GC-2 and TM4 murine cells, with respect to the control, determined a nearly 50% of death, that doubles the effect observed in Empty Vector (EV) transfected H2O2-treated cells. The enforced expression of myc CCDC6T434A in H2O2 treated CCDC6 null GC-2 and TM4 cells, compared to control, resulted in 33% of death, significantly lower than CCDC6 WT effect (p<0.01) and almost comparable to the effect observed in same cells upon H2O2 treatment and EV transfection. Indeed, Z-VAD-fmk, Ferrostatin-1 and Deferoxamine treatment insignificantly rescued the H2O2-induced cell death in CCDC6T434A H2O2 treated CCDC6 null GC-2 and TM4 cells (p >0.05). [15] (Fig. 4 C, D).

In conclusion, the loss of CCDC6 or the overexpression of CCDC6 point mutant protected the testicular murine and human cancer cells from reactive oxygen species by limiting the regulated cell death by apoptosis and ferroptosis.

### 3.5 The loss of function of CCDC6 increases resistance to oxidative DNA damage by enhancing the xCT/SLC7A11 expression and impairing ferroptosis

**Figure 5:**
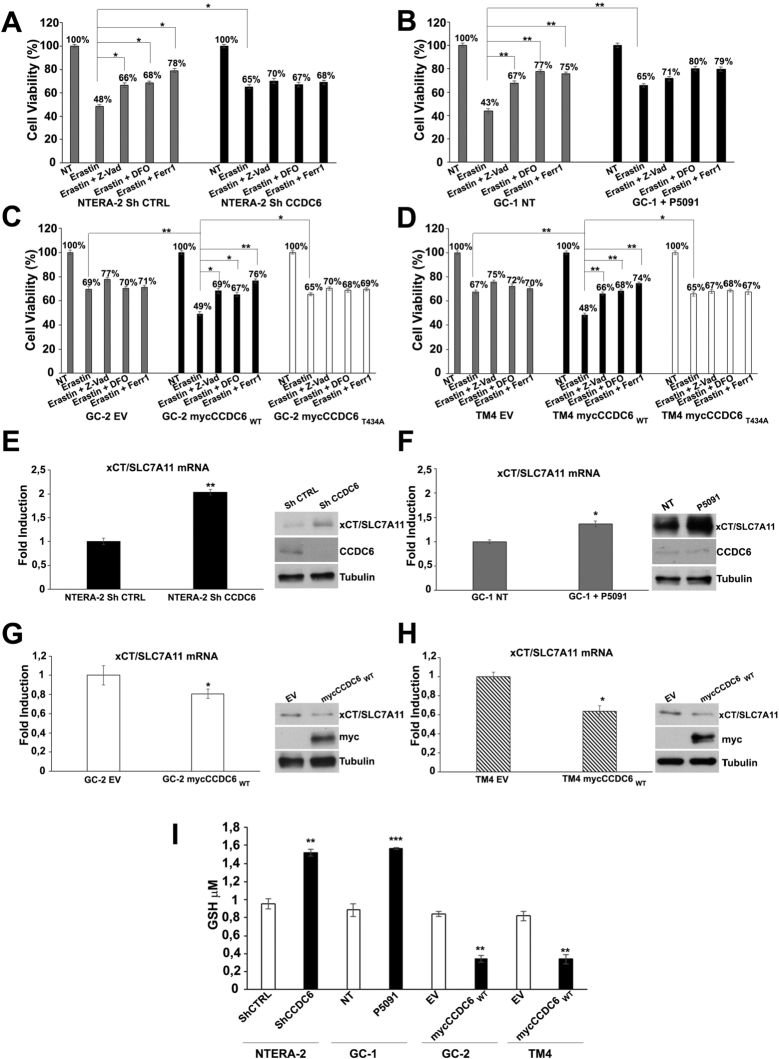
CCDC6 loss associates with enhanced levels of the xCT/SLC7A11 cystine antiporter at RNA and protein levels in human NTERA-2 Embryonal Carcinoma and in murine testicular cells. Bar graphs showing cell viability of CCDC6-silenced (**A**) human NTERA-2 cells (upon transient transfection with shCCDC6 or control, shCTRL), (**B**) murine spermatogonia GC-1 cells (upon addition of P5091 or vehicle) and of CCDC6 natively null (**C**) murine spermatocytes GC-2 and (**D**) murine Sertoli TM4 cells (restored with empty vector (EV), as control, and with CCDC6 wt or CCDC6T434A mutant), treated with 20 μM erastin combined with 5 μM Z-VAD-fmk (Z-VAD), 2 μM ferrostatin-1 (Ferr-1) or 100 μM deferoxamine (DFO). (**E-H**) xCT/SLC7A11 relative expression was assessed by RealTime PCR in the human NTERA-2 EC cells (upon transient transfection with shCCDC6 or control, shCTRL), in the murine GC-1 (upon addition of P5091 or vehicle), and in CCDC6 natively null GC-2 and TM4 cells (restored with CCDC6wt). Western Blot analysis of xCT/SLC7A11 and CCDC6 expression levels were detected in the same cell populations. Anti-tubulin immunoblots are shown as loading control. Error bars are mean ± s.d., of 3 independent repeats. (**I**) Bar graph showing intracellular GSH levels in indicated cells. Statistical significance was verified by 2-tailed Student's t-test (* p <0.05; ** p <0.01 and *** p <0.001). In **A**, **B**, **C**, and **D** percentage numbers have added to each histogram, as shown.

Ferroptosis is an iron-regulated caspase-independent cell death modality that was first reported in 2012 [5]. It is implicated in lipid-peroxidation induced cell death in a series of diseases, including cancer. Ferroptosis is initiated through a loss of activity of the lipid repair enzyme, glutathione peroxidase 4 (GPX4) and/or an inhibition of cystine uptake, which causes a depletion of glutathione (GSH) and determines a lethal accumulation of reactive oxygen metabolites. Solute carrier family 7 member 11 (SLC7A11, also called xCT) is the major transporter of extracellular cystine [46], [47], [48], and cystine depletion or drugs that block xCT-mediated cystine uptake, such as erastin, induce ferroptosis [5], [49]. The genetic depletion of CCDC6, by shCCDC6, made the NTERA-2 cells resistant to erastin-induced ferroptosis (Fig. 5 A), as well as the CCDC6 chemical silencing, by P5091, protected the spermatogonia GC-1 cells, from the erastin induced cell death, compared to the control cells (Fig. 5 B). Similar results were retrieved upon CCDC6 chemical silencing with P5091 in NTERA-2 cells (Supplementary Figure 2 C) and following CCDC6 depletion by shRNA in GC-1 murine cells (Supplementary Figures 2 D).

**Figure 6:**
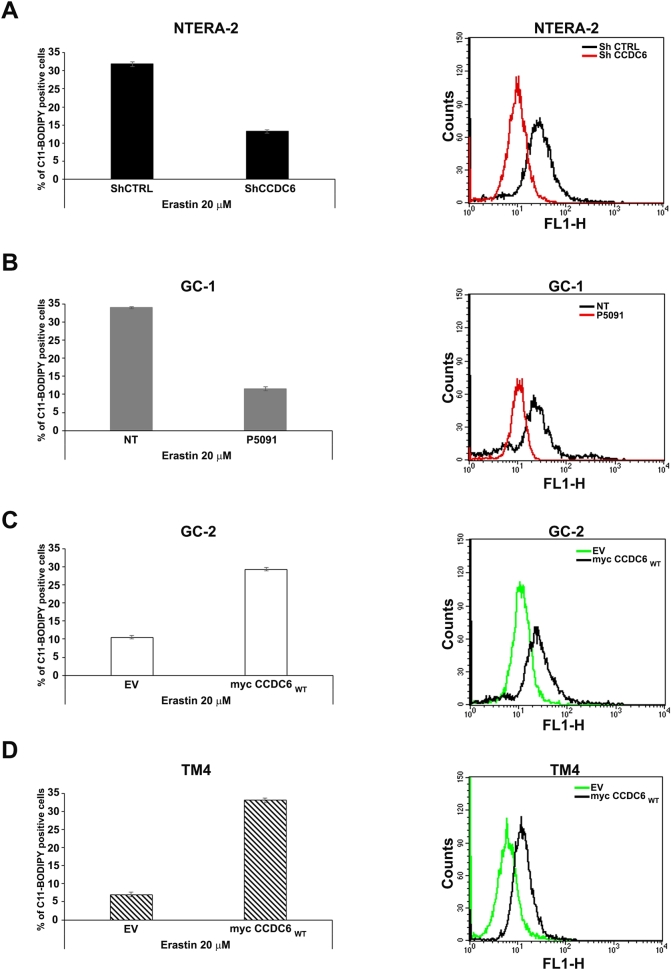
CCDC6 loss associates with reduced lipid peroxidation levels in human NTERA-2 Embryonal Carcinoma and in murine testicular cells. (**A**, **B**, **C**, **D**, **left**) Bar graphs show the percentage of C11-BODIPY positive cells after treatment with 20 μM of Erastin. (**A**, **B**, **C**, **D**, **right**) Histogram plots generated after flow cytometer analyses, show the overlay of fluorescence in each cell line. Error bars are mean ± s.d., of 3 independent repeats.

In control cells (NTERA-2 shCTRL transfected cells and in vehicle-treated GC-1 cells) the erastin-induced cell death was largely suppressed by the ferroptosis inhibitor ferrostatin or the iron chelator deferoxamine (DFO), while barely inhibited by the apoptosis inhibitor Z-VAD-fmk (Fig. 5 A, B). Thus, the CCDC6 deficiency made the testis cells more resistant to erastin-induced ferroptosis. Importantly, re-expression of the CCDC6wt, but not of its T434A mutant, in natively CCDC6 null GC-2 and TM4 cells restored the ferroptosis sensitivity (Fig. 5 C, D). The xCT/SLC7A11 expression, upregulated in response to ROS-inducing agents, has been implicated in promoting tumourigenesis through its antioxidant function, and is directly repressed by p53 and BAP1 [50], [51], [52], [53], [54], [55]. Moreover, the genetic or pharmacological inhibition of xCT/SLC7A11, such as with erastin, hold promise as a therapeutic strategy in several tumours [56], [57]. In order to investigate whether CCDC6 promotes ferroptosis by modulating xCT/SLC7A11 expression and activity, in natively CCDC6 null testicular cells GC-2 and TM4, we re-expressed CCDC6wt observing a reduction of the xCT/SLC7A11 expression at mRNA and protein level. A decrease of GSH levels was detected following CCDC6 re-expression, too (Fig. 5 I). Strikingly, the transient CCDC6 silencing in GC-1 and in NTERA-2 embryonal carcinoma cells, resulted associated with an increase of xCT/SLC7A11 at mRNA and protein level. Indeed, an increase of GSH levels was also observed after the CCDC6 depletion in the same cells (Fig. 5 E-I). Furthermore, since ferroptosis associates with enhanced levels of lipid peroxidation (lipid ROS) and in order to confirm that the CCDC6 deficiency may lead to lipid ROS tolerance and ferroptosis evasion, we analyzed the levels of lipid peroxidation in our cellular models. In the CCDC6 null (TM4 and GC-2) cells, that show appreciable levels of the xCT/SLC7A11 channel, we observed low levels of lipid peroxidation assessed by flow cytometry. The transient CCDC6 depletion in the NTERA-2 and GC-1 cells gave comparable results (Fig. 6).

**Figure 7:**
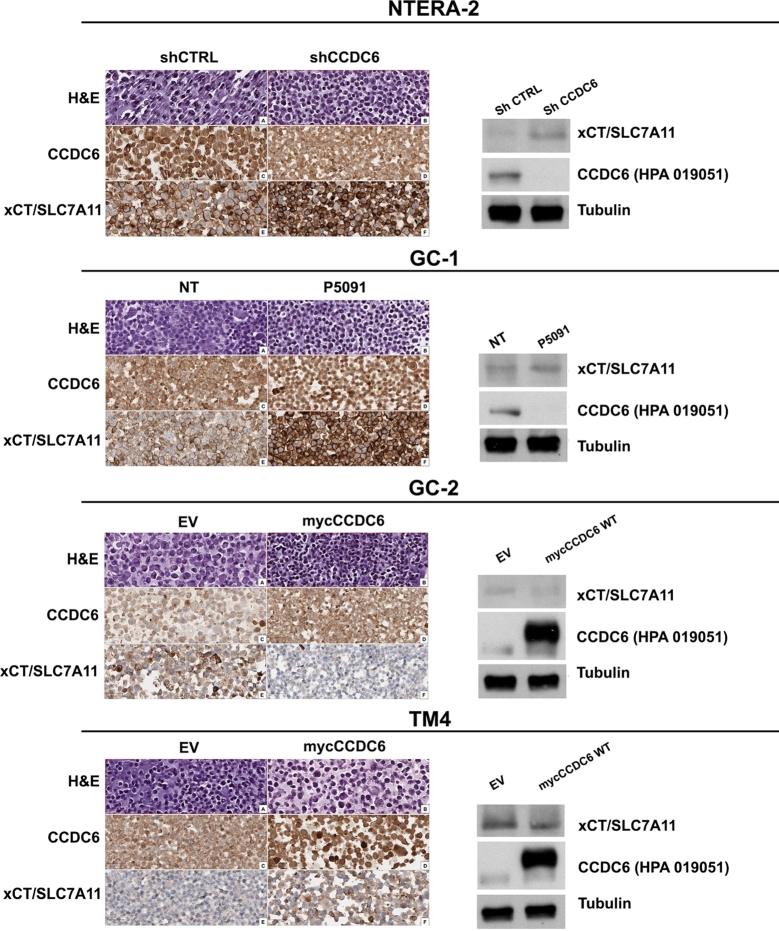
Immunocytochemical analysis of CCDC6 and xCT/SLC7A11 proteins expression in cell block preparation of NTERA-2, GC-1, GC-2, and TM4 cells, wild type or engineered for CCDC6 silencing or re-expression, as indicated. The levels of CCDC6 (detected by HPA019051 antibody) and xCT/SLC7A11 proteins were assessed by Western Blot analysis in the same cell populations. Anti-tubulin is shown as loading control.

Finally, The immunocytochemical staining with anti-CCDC6 (HPA019051) and anti-xCT/SLC7A11 antibodies in the Embryonal Carcinoma NTERA-2 cells and in murine testicular cells, wild type or engineered for CCDC6 depletion or overexpression, showed that the expression of CCDC6 and xCT/SLC7A11 proteins inversely correlated, as also appreciated by western blot analysis performed with the same antibodies (Fig. 7).

## 4 Discussion

Testicular germ cell tumours represent one of the most common malignancy in young men and include seminoma and non-seminatous tumours. Among the last, embryonal carcinomas arise from the stem cell component of the testis, present high invasiveness, and show an increased risk of relapse in the mixed forms of tumours. Even though an increased incidence of TGCTs is recorded, the mortality remains stable, as more than 90% of cure rate is achieved by surgery and/or chemotherapy. However, in the era of targeted therapies no significant improvements have been obtained for resistant tumours [58].

Spermatogenesis is a complex process in which spermatogonia stem cells self-renew and differentiate into spermatozoa under coordination of the testicular microenvironment provided by Sertoli cells, which represents the only somatic cells located inside the seminiferous tubule. Sertoli cells are the major supportive germ cells that regulate spermatogenic cells via a number of paracrine factors. Germ cells and Sertoli cells interactions are important for male fertility; however, an unbalanced cross talk between this cell population may also lead to neoplastic transformation. The germinal and somatic cells, continuously exposed to oxidative DNA damage, are controlled by cell death pathways of apoptosis and ferroptosis [25], the last envisaged as emerging mechanism of carcinogenesis.

In these cell systems we focused our attention on CCDC6, a tumour suppressor gene, involved in apoptosis and in DNA damage response, and known to be functionally lost upon gene translocations, somatic mutations and altered protein levels in several tumours [16]. Here we report that in EC cells the depletion of CCDC6 determined an impairment of DNA DSBs repair by homologous recombination and PARP inhibitor sensitivity. We also observed that the CCDC6 silencing in the EC cells determined tolerance to hydrogen-peroxide and to erastin, an inhibitor of the xCT/SLC7A11 cystine antiporter, by limiting apoptosis and ferroptosis regulated cell death pathways. The tolerance to reactive oxygen species (ROS) was also revealed in the spontaneously null Sertoli and spermatocytes cells, suggesting that different levels of CCDC6 expression during the spermatocytic differentiation process could reflect the different levels of control needed for the mitotically proliferating testicular cells rather than during meiotic recombination, later during the differentiation process, where CCDC6 could probably induce inadequate regulated cell death and undermine spermatogenesis. Further, spermatozoa are subjected to enormous oxidative stress during their development, and the role of CCDC6 protein in protecting DNA integrity in these cells is emerging. Nevertheless, functional roles in reproductive physiology and pathology ascribed to the family of proteins containing a coiled-coil domain (CCDC) has been recently reviewed [59].

In embryonal carcinoma and in the testicular cells, following exposure to the oxidative damage, we report that the loss of CCDC6 determined tolerance to reactive oxygen species affecting apoptosis and ferroptosis. The involvement of ferroptosis in testicular cells has been already reported in Sertoli cells, in response to hypoxia and re-oxigenation processes [25].

However, the possibilities to modulate the response to ROS depending on the levels or activity of CCDC6 has not been explored before. At the molecular level we observed that the CCDC6 depletion, in NTERA-2 EC and in the GC-1 spermatogonia cells, was associated with the induction of the xCT/SLC7A11 cystine antiporter expression which, by enhancing the GSH levels, contributes to detoxify from ROS accumulation interfering with the ferroptosis pathway. Nonetheless, the CCDC6 re-expression in CCDC6-spontaneously null TM4 (Sertoli) and GC-2 (Spermatocytes) cell lines resulted associated with reduced xCT/SLC7A11 expression at mRNA and protein levels leading to ferroptosis. In support of that, the adult mouse testis, at immunohistochemistry, showed a nearly complementary xCT/SLC7A11 and CCDC6 pattern of expression in seminiferous tubules (Fig. 1 A-D).

Ferroptosis is a newly discovered cell death mechanism, which is characterized by iron-dependent accumulation of lethal lipid peroxidation. It is known that ferroptosis is distinct from apoptosis, necrosis and autophagy at genetic, biochemical and morphological levels. To date, emerging evidence has shown the potential of triggering ferroptosis for cancer therapy [6].

Indeed, ferroptosis has been reported as a ubiquitous and effective type of cell death induced by oxidative stress and described in Sertoli suggesting novel insight into the application of cytoprotection in testicular I/R damage-induced cell loss [25]. Interestingly, the in vitro conditional expression of CCDC6 dominant negative mutants protected cancer cells from oxidative stress, also impairing the stress-induced cell death [17]. In response to genotoxic stress CCDC6 translocates to the nucleus where it participates to the homologous recombination machinery by binding the protein phosphatase PP4c and regulating the H2AX phosphorylation [18]. Moreover, preclinical studies indicates that the attenuation of CCDC6 in lung, pleural, prostate and bladder cancer cells confer sensitivity to inhibitors of PARP1/2 [19], [20], [35], [36].

Strikingly, here we report that in testicular tumours the CCDC6 deficiency specifically associated to impairment of homologous recombination of DNA DSBs which associates to PARP inhibitors sensitivity. Then, we believe that CCDC6 could represent a useful predictive and reliable biomarker for the use of PARP inhibitors in tumour testis. These observations might have clinical relevance as currently, two phase II clinical trials are evaluating the potential of PARP inhibition in relapsed or refractory testicular tumours, either as single agent (NCT02533765) or combined with gemcitabine and carboplatin (NCT02860819).

**Figure S3:**
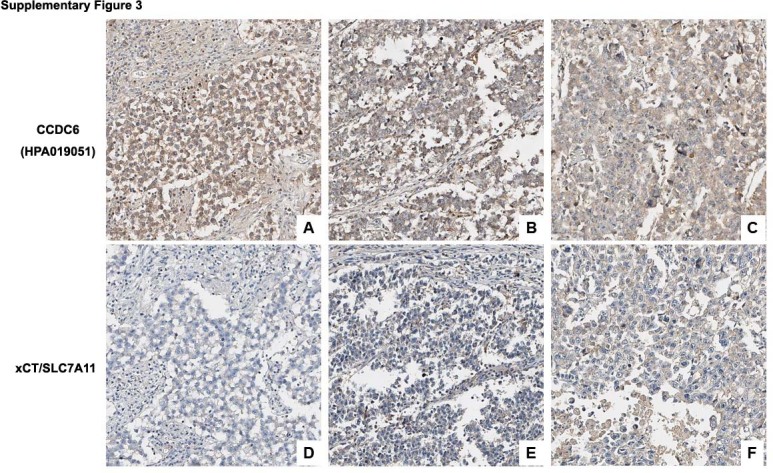
IHC analysis of CCDC6 and xCT/SLC7A11 proteins expression in FFPE samples of seminoma (**A**, **D**), anaplastic seminoma (**B**, **E**), and embryonal carcinoma (**C**, **F**). Magnification 20x on digitally acquired WSI.

In normal cells CCDC6 might inhibit cystine uptake by repressing xCT/SLC7A11 expression and leading to elevated lipid peroxidation and ferroptosis. Conversely, in cancers which carry CCDC6 mutated forms, or that have lost CCDC6 expression, cancer cells may lose their abilities to repress xCT/SLC7A11 and to promote ferroptosis. At immunohistochemistry (IHC) the evaluation of a pilot subset of primary testicular tumours, revealed (Supplementary Figure 3) that CCDC6 staining inversely correlated with the xCT/SLC7A11 expression levels, supporting our *in vitro* evidence (Fig. 7).

Moreover, CCDC6 is known to act as negative transcription regulator of CREB family members, including CREB1 [60] and the Activating Transcription Factor 4 (ATF4) (Cerrato A. et al. personal communication). Therefore, as the xCT promoter regulation depends on ETS1 and also on ATF4 transcription factors, we can hypothesize that CCDC6 loss or functional impairment may lead to a continuous and uncontrolled ATF4 activity which could end up in an enhanced xCT transcription, increased GSH levels, and evasion from the regulated cell death pathways of apoptosis and ferroptosis, the last observed here for the first time. [24], [60], [61], [62].

Finally, a better understanding of the mechanisms of ROS tolerance, could aid to draw novel therapeutic approaches, by taking advantage of xCT/SLC7A11 channel inhibitors and/or ferroptosis inhibitors. In the future these drugs can be utilized in resistant TGCTs in association with targeted approaches which could re-sensitize the tumours to the standard therapies [63].

## 5 Conclusions

In conclusion, the data we reported suggest that the loss of function of CCDC6, which helps to elude the genotoxic effects of the reactive oxygen species, might highly contribute to the carcinogenetic process in testis. The ability to evade the regulated cell death pathways of apoptosis and ferroptosis, as emerged from our data, could provide support for proposing novel potential targets for the treatment of resistant tumour of the testis, also based on the increasing understanding of the most recent advancement in ferroptosis.

## Declarations

### Author contribution statement

Francesco Morra: Conceived and designed the experiments; Performed the experiments; Analyzed and interpreted the data; Contributed reagents, materials, analysis tools or data; Wrote the paper.

Francesco Merolla, Paolo Chieffi: Conceived and designed the experiments; Analyzed and interpreted the data; Contributed reagents, materials, analysis tools or data; Wrote the paper.

Federica Zito Marino, Rosaria Catalano: Performed the experiments; Contributed reagents, materials, analysis tools or data.

Renato Franco: Analyzed and interpreted the data; Contributed reagents, materials, analysis tools or data.

Angela Celetti: Conceived and designed the experiments; Analyzed and interpreted the data; Wrote the paper.

### Funding statement

This work was supported by POR Campania FESR 2014-2020 “SATIN” grant.

### Data availability statement

Data will be made available on request.

### Declaration of interests statement

The authors declare no conflict of interest.

### Additional information

Supplementary content related to this article has been published online at https://doi.org/10.1016/j.heliyon.2021.e08399.

No additional information is available for this paper.

## Appendix A Supplementary material

The following Supplementary material is associated with this article:

Supplementary Figure 4.pdf The uncropped versions of Figure 1E, Figure 2, Figure 3B, Figure 4, Figure 5, Figure 7, Supplementary Figure 1B and Supplementary Figure 2 are shown. Supplementary Figure 4.pdf

## References

[1] Moch H., Cubilla A.L., Humphrey P.A., Reuter V.E., Ulbright T.M. (2016). The 2016 WHO classification of tumours of the urinary system and male genital organs—part a: renal, penile, and testicular tumours. European Urology.

[2] Shanmugalingam T., Soultati A., Chowdhury S., Rudman S., Van Hemelrijck M. (2013). Global incidence and outcome of testicular cancer. Clinical Epidemiology.

[3] Bromfield E.G., Walters J.L.H., Cafe S.L., Bernstein I.R., Stanger S.J., Anderson A.L., Aitken R.J., McLaughlin E.A., Dun M.D., Gadella B.M., Nixon B. (2019). Differential cell death decisions in the testis: evidence for an exclusive window of ferroptosis in round spermatids. MHR: Basic science of reproductive medicine.

[4] Tang D., Kang R., Berghe T.V., Vandenabeele P., Kroemer G. (2019). The molecular machinery of regulated cell death. Cell Research.

[5] Dixon S., Lemberg K., Lamprecht M., Skouta R., Zaitsev E., Gleason C., Patel D., Bauer A., Cantley A., Yang W., Morrison B., Stockwell B. (2012). Ferroptosis: an iron-dependent form of nonapoptotic cell death. Cell.

[6] Tang D., Chen X., Kang R., Kroemer G. (2021). Ferroptosis: molecular mechanisms and health implications. Cell Research.

[7] Litchfield K., Summersgill B., Yost S., Sultana R., Labreche K., Dudakia D., Renwick A., Seal S., Al-Saadi R., Broderick P., Turner N.C., Houlston R.S., Huddart R., Shipley J., Turnbull C. (2015). Whole-exome sequencing reveals the mutational spectrum of testicular germ cell tumours. Nature Communications.

[8] Kemmer K., Corless C.L., Fletcher J.A., McGreevey L., Haley A., Griffith D., Cummings O.W., Wait C., Town A., Heinrich M.C. (2004). KIT mutations are common in testicular seminomas. The American Journal of Pathology.

[9] Litchfield K., Levy M., Huddart R.A., Shipley J., Turnbull C. (2016). The genomic landscape of testicular germ cell tumours: from susceptibility to treatment. Nature Reviews Urology.

[10] Olie R.A., Looijenga L.H., Boerrigter L., Top B., Rodenhuis S., Langeveld A., Mulder M.P., Oosterhuis J.W. (1995). N- and KRAS mutations in primary testicular germ cell tumors: incidence and possible biological implications. Genes, Chromosomes & Cancer.

[11] Cutcutache I., Suzuki Y., Tan I.B., Ramgopal S., Zhang S., Ramnarayanan K., Gan A., Lee H.H., Tay S.T., Ooi A., Ong C.K., Bolthouse J.T., Lane B.R., Anema J.G., Kahnoski R.J., Tan P., Teh B.T., Rozen S.G. (2015). Exome-wide sequencing shows low mutation rates and identifies novel mutated genes in seminomas. European Urology.

[12] Shen H., Shih J., Hollern D.P., Wang L., Bowlby R., Tickoo S.K., Thorsson V., Mungall A.J., Newton Y., Hegde A.M., Armenia J., Sánchez-Vega F., Pluta J., Pyle L.C., Mehra R., Reuter V.E., Godoy G., Jones J., Shelley C.S., Feldman D.R., Vidal D.O., Lessel D., Kulis T., Cárcano F.M., Leraas K.M., Lichtenberg T.M., Brooks D., Cherniack A.D., Cho J., Heiman D.I., Kasaian K., Liu M., Noble M.S., Xi L., Zhang H., Zhou W., ZenKlusen J.C., Hutter C.M., Felau I., Zhang J., Schultz N., Getz G., Meyerson M., Stuart J.M., Akbani R., Wheeler D.A., Laird P.W., Nathanson K.L., Cortessis V.K., Hoadley K.A., Wang L., Xi L., Wheeler D., Hughes D., Covington K., Jayaseelan J.C., Korchina V., Lewis L., Hu J., Doddapaneni H., Muzny D., Gibbs R., Hoadley K.A., Hollern D., Vincent B.G., Chai S., Smith C.C., Auman J.T., Shi Y., Meng S., Skelly T., Tan D., Veluvolu U., Mieczkowski P.A., Jones C.D., Wilkerson M.D., Balu S., Bodenheimer T., Hoyle A.P., Jefferys S.R., Mose L.E., Simons J.V., Soloway M.G., Roach J., Parker J.S., Hayes D.N., Perou C.M., Shih J., Cherniack A.D., Meyerson M., Saksena G., Cibulskis C., Schumacher S.E., Beroukhim R., Gabriel S.B., Bowlby R., Mungall A.J., Brooks D., Kasaian K., Ally A., Balasundaram M., Carlsen R., Cheung D., Chuah E., Dhalla N., Holt R.A., Jones S.J., Ma Y., Mayo M., Moore R.A., Robertson A.G., Schein J.E., Sipahimalani P., Tam A., Thiessen N., Wong T., Marra M.A., Shen H., Zhou W., Laird P.W., Weisenberger D.J., Van Den Berg D.J., Lai P.H., Berrios M., Holbrook A., Bootwalla M.S., Maglinte D.T., Armenia J., Sánchez-Vega F., Schultz N., Chakravarty D., Gao J., Heins Z., Kundra R., Ochoa A., Liu M., Sander C., Ladanyi M., Thorsson V., Radenbaugh A.J., Newton Y., Stuart J.M., Cho J., Heiman D.I., Noble M.S., Zhang H., Getz G., Gehlenborg N., Saksena G., Voet D., Lin P., Frazer S., Kim J., Lawrence M.S., Meier S., Defreitas T., Chin L., Hegde A.M., Akbani R., Weinstein J.N., Liu W., Mills G.B., Lu Y., Pyle L.C., Pluta J., Nathanson K.L., Tickoo S.K., Reuter V.E., Mehra R., Looijenga L., Bryce A.H., Cárcano F.M., Carvalho A.L., Cortessis V.K., Feldman D., Godoy G., Ittmann M., Jones J., Kulis T., Lerner S., Lessel D., Nathanson K.L., Shelley C.S., Vidal D.O., Leraas K.M., Lichtenberg T.M., Bowen J., Gastier-Foster J.M., Gerken M., Helsel C., Ramirez N.C., Wise L., Zmuda E., Cottingham S., Chesla D., Saller C., Tarvin K., Lopes L.F., Scapulatempo-Neto C., Aredes N.D., Oosterhuis W., Gillis A., Stoop H., Eijkenboom W., Sandusky G., Martin S.E., Aron M., Daneshmand S., Djaladat H., Quinn D., Dorff T., Lennerz J.K., Thorne L.B., Gamulin M., Kastelan Z., Hudolin T., Kubisch C., Boice L., Huang M., Perou A.H., Rathmell W.K., Pihl T., Wan Y., Sun Q., Naresh R., Chudamani S., Liu J., Lolla L., Wu Y., Ferguson M.L., Zenklusen J.C., Felau I., Zhang J.J., Sheth M., Demchok J.A., Yang L., Wang Z., Tarnuzzer R., Hutter C.M., Sofia H.J., Davidsen T.M. (2018). Integrated molecular characterization of testicular germ cell tumors. Cell Reports.

[13] Einhorn L.H. (1990). Treatment of testicular cancer: a new and improved model. Journal of Clinical Oncology.

[14] Cavallo F., Graziani G., Antinozzi C., Feldman D.R., Houldsworth J., Bosl G.J., Chaganti R.S.K., Moynahan M.E., Jasin M., Barchi M. (2012). Reduced proficiency in homologous recombination underlies the high sensitivity of embryonal carcinoma testicular germ cell tumors to cisplatin and poly (ADP-ribose) polymerase inhibition. PLoS ONE.

[15] Merolla F., Pentimalli F., Pacelli R., Vecchio G., Fusco A., Grieco M., Celetti A. (2007). Involvement of H4(D10S170) protein in ATM-dependent response to DNA damage. Oncogene.

[16] Cerrato A., Merolla F., Morra F., Celetti A. (2018). CCDC6: the identity of a protein known to be partner in fusion: CCDC6 a partner in fusion. International Journal of Cancer.

[17] Celetti A., Cerrato A., Merolla F., Vitagliano D., Vecchio G., Grieco M. (2004). H4(D10S170), a gene frequently rearranged with RET in papillary thyroid carcinomas: functional characterization. Oncogene.

[18] Merolla F., Luise C., Muller M.T., Pacelli R., Fusco A., Celetti A. (2012). Loss of CCDC6, the first identified RET partner gene, affects pH2AX S139 levels and accelerates mitotic entry upon DNA damage. PLoS ONE.

[19] Visconti R., Morra F., Guggino G., Celetti A. (2017). The between now and then of lung cancer chemotherapy and immunotherapy. International Journal of Molecular Sciences.

[20] Morra F., Merolla F., D'Abbiero D., Ilardi G., Campione S., Monaco R., Guggino G., Ambrosio F., Staibano S., Cerrato A., Visconti R., Celetti A. (2019). Analysis of CCDC6 as a novel biomarker for the clinical use of PARP1 inhibitors in malignant pleural mesothelioma. Lung Cancer.

[21] Criscuolo D., Morra F., Giannella R., Cerrato A., Celetti A. (2019). Identification of novel biomarkers of homologous recombination defect in DNA repair to predict sensitivity of prostate cancer cells to PARP-inhibitors. International Journal of Molecular Sciences.

[22] Morra F., Merolla F., Criscuolo D., Insabato L., Giannella R., Ilardi G., Cerrato A., Visconti R., Staibano S., Celetti A. (2019). CCDC6 and USP7 expression levels suggest novel treatment options in high-grade urothelial bladder cancer. Journal of Experimental & Clinical Cancer Research.

[23] Sato H., Tamba M., Ishii T., Bannai S. (1999). Cloning and expression of a plasma membrane cystine/glutamate exchange transporter composed of two distinct proteins. Journal of Biological Chemistry.

[24] Lim J.K.M., Delaidelli A., Minaker S.W., Zhang H.-F., Colovic M., Yang H., Negri G.L., von Karstedt S., Lockwood W.W., Schaffer P., Leprivier G., Sorensen P.H. (2019). Cystine/glutamate antiporter xCT (SLC7A11) facilitates oncogenic RAS transformation by preserving intracellular redox balance. Proceedings of the National Academy of Sciences.

[25] Li L., Hao Y., Zhao Y., Wang H., Zhao X., Jiang Y., Gao F. (2018). Ferroptosis is associated with oxygen-glucose deprivation/reoxygenation-induced Sertoli cell death. International Journal of Molecular Medicine.

[26] Hofmann M.C., Hess R.A., Goldberg E., Millán J.L. (1994). Immortalized germ cells undergo meiosis in vitro. Proceedings of the National Academy of Sciences of the United States of America.

[27] Esposito F., Boscia F., Gigantino V., Tornincasa M., Fusco A., Franco R., Chieffi P. (2012). The high-mobility group A1-estrogen receptor *β* nuclear interaction is impaired in human testicular seminomas. Journal of Cellular Physiology.

[28] Rossi M., Colecchia D., Ilardi G., Acunzo M., Nigita G., Sasdelli F., Celetti A., Strambi A., Staibano S., Croce C.M., Chiariello M. (2016). MAPK15 upregulation promotes cell proliferation and prevents DNA damage in male germ cell tumors. Oncotarget.

[29] Jasin M. (1996). Genetic manipulation of genomes with rare-cutting endonucleases. Trends in Genetics.

[30] Russo D., Merolla F., Mascolo M., Ilardi G., Romano S., Varricchio S., Napolitano V., Celetti A., Postiglione L., Di Lorenzo P.P., Califano L., Dell'Aversana G.O., Astarita F., Romano M.F., Staibano S. (2 2017). FKBP51 immunohistochemical expression: a new prognostic biomarker for OSCC?. International Journal of Molecular Sciences.

[31] Martino F., Varricchio S., Russo D., Merolla F., Ilardi G., Mascolo M., Dell'aversana G.O., Califano L., Toscano G., De Pietro G., Frucci M., Brancati N., Fraggetta F., Staibano S. (2020). A machine-learning approach for the assessment of the proliferative compartment of solid tumors on hematoxylin-eosin-stained sections. Cancers.

[32] Bankhead P., Loughrey M.B., Fernández J.A., Dombrowski Y., McArt D.G., Dunne P.D., McQuaid S., Gray R.T., Murray L.J., Coleman H.G., James J.A., Salto-Tellez M., Hamilton P.W. (2017). Qupath: open source software for digital pathology image analysis. Scientific Reports.

[33] Khan S., Omar T., Michelow P. (2012). Effectiveness of the cell block technique in diagnostic cytopathology. Journal of Cytology.

[34] Turnbull A.P., Ioannidis S., Krajewski W.W., Pinto-Fernandez A., Heride C., Martin A.C.L., Tonkin L.M., Townsend E.C., Buker S.M., Lancia D.R., Caravella J.A., Toms A.V., Charlton T.M., Lahdenranta J., Wilker E., Follows B.C., Evans N.J., Stead L., Alli C., Zarayskiy V.V., Talbot A.C., Buckmelter A.J., Wang M., McKinnon C.L., Saab F., McGouran J.F., Century H., Gersch M., Pittman M.S., Marshall C.G., Raynham T.M., Simcox M., Stewart L.M.D., McLoughlin S.B., Escobedo J.A., Bair K.W., Dinsmore C.J., Hammonds T.R., Kim S., Urbé S., Clague M.J., Kessler B.M., Komander D. (2017). Molecular basis of USP7 inhibition by selective small-molecule inhibitors. Nature.

[35] Morra F., Luise C., Merolla F., Poser I., Visconti R., Ilardi G., Paladino S., Inuzuka H., Guggino G., Monaco R., Colecchia D., Monaco G., Cerrato A., Chiariello M., Denning K., Claudio P.P., Staibano S., Celetti A. (2015). FBXW7 and USP7 regulate CCDC6 turnover during the cell cycle and affect cancer drugs susceptibility in NSCLC. Oncotarget.

[36] Malapelle U., Morra F., Ilardi G., Visconti R., Merolla F., Cerrato A., Napolitano V., Monaco R., Guggino G., Monaco G., Staibano S., Troncone G., Celetti A. (2017). USP7 inhibitors, downregulating CCDC6, sensitize lung neuroendocrine cancer cells to PARP-inhibitor drugs. Lung Cancer.

[37] Morra F., Merolla F., Napolitano V., Ilardi G., Miro C., Paladino S., Staibano S., Cerrato A., Celetti A. (2017). The combined effect of USP7 inhibitors and PARP inhibitors in hormone-sensitive and castration-resistant prostate cancer cells. Oncotarget.

[38] Morra F., Luise C., Visconti R., Staibano S., Merolla F., Ilardi G., Guggino G., Paladino S., Sarnataro D., Franco R., Monaco R., Zitomarino F., Pacelli R., Monaco G., Rocco G., Cerrato A., Linardopoulos S., Muller M.T., Celetti A. (2015). New therapeutic perspectives in CCDC6 deficient lung cancer cells. International Journal of Cancer.

[39] Cerrato A., Morra F., Celetti A. (2016). Use of poly ADP-ribose polymerase [PARP] inhibitors in cancer cells bearing DDR defects: the rationale for their inclusion in the clinic. Journal of Experimental & Clinical Cancer Research.

[40] Chou T.-C. (2006). Theoretical basis, experimental design, and computerized simulation of synergism and antagonism in drug combination studies. Pharmacological Reviews.

[41] Cerrato A., Visconti R., Celetti A. (2018). The rationale for druggability of CCDC6-tyrosine kinase fusions in lung cancer. Molecular Cancer.

[42] Cerrato A., Morra F., Di Domenico I., Celetti A. (12 2019). NSCLC mutated isoforms of CCDC6 affect the intracellular distribution of the wild type protein promoting cisplatinum resistance and PARP inhibitors sensitivity in lung cancer cells. Cancers.

[43] Lee Y.-S., Lee D.-H., Choudry H.A., Bartlett D.L., Lee Y.J. (2018). Ferroptosis-induced endoplasmic reticulum stress: cross-talk between ferroptosis and apoptosis. Molecular Cancer Research.

[44] Galluzzi L., Vitale I., Aaronson S.A., Abrams J.M., Adam D., Agostinis P., Alnemri E.S., Altucci L., Amelio I., Andrews D.W., Annicchiarico-Petruzzelli M., Antonov A.V., Arama E., Baehrecke E.H., Barlev N.A., Bazan N.G., Bernassola F., Bertrand M.J.M., Bianchi K., Blagosklonny M.V., Blomgren K., Borner C., Boya P., Brenner C., Campanella M., Candi E., Carmona-Gutierrez D., Cecconi F., Chan F.K.-M., Chandel N.S., Cheng E.H., Chipuk J.E., Cidlowski J.A., Ciechanover A., Cohen G.M., Conrad M., Cubillos-Ruiz J.R., Czabotar P.E., D'Angiolella V., Dawson T.M., Dawson V.L., De Laurenzi V., De Maria R., Debatin K.-M., DeBerardinis R.J., Deshmukh M., Di Daniele N., Di Virgilio F., Dixit V.M., Dixon S.J., Duckett C.S., Dynlacht B.D., El-Deiry W.S., Elrod J.W., Fimia G.M., Fulda S., García-Sáez A.J., Garg A.D., Garrido C., Gavathiotis E., Golstein P., Gottlieb E., Green D.R., Greene L.A., Gronemeyer H., Gross A., Hajnoczky G., Hardwick J.M., Harris I.S., Hengartner M.O., Hetz C., Ichijo H., Jäättelä M., Joseph B., Jost P.J., Juin P.P., Kaiser W.J., Karin M., Kaufmann T., Kepp O., Kimchi A., Kitsis R.N., Klionsky D.J., Knight R.A., Kumar S., Lee S.W., Lemasters J.J., Levine B., Linkermann A., Lipton S.A., Lockshin R.A., López-Otín C., Lowe S.W., Luedde T., Lugli E., MacFarlane M., Madeo F., Malewicz M., Malorni W., Manic G., Marine J.-C., Martin S.J., Martinou J.-C., Medema J.P., Mehlen P., Meier P., Melino S., Miao E.A., Molkentin J.D., Moll U.M., Muñoz-Pinedo C., Nagata S., Nuñez G., Oberst A., Oren M., Overholtzer M., Pagano M., Panaretakis T., Pasparakis M., Penninger J.M., Pereira D.M., Pervaiz S., Peter M.E., Piacentini M., Pinton P., Prehn J.H., Puthalakath H., Rabinovich G.A., Rehm M., Rizzuto R., Rodrigues C.M., Rubinsztein D.C., Rudel T., Ryan K.M., Sayan E., Scorrano L., Shao F., Shi Y., Silke J., Simon H.-U., Sistigu A., Stockwell B.R., Strasser A., Szabadkai G., Tait S.W., Tang D., Tavernarakis N., Thorburn A., Tsujimoto Y., Turk B., Vanden Berghe T., Vandenabeele P., Vander Heiden M.G., Villunger A., Virgin H.W., Vousden K.H., Vucic D., Wagner E.F., Walczak H., Wallach D., Wang Y., Wells J.A., Wood W., Yuan J., Zakeri Z., Zhivotovsky B., Zitvogel L., Melino G., Kroemer G. (2018). Molecular mechanisms of cell death: recommendations of the Nomenclature Committee on Cell Death 2018. Cell Death & Differentiation.

[45] Zilka O., Shah R., Li B., Friedmann Angeli J.P., Griesser M., Conrad M., Pratt D.A. (2017). On the mechanism of cytoprotection by ferrostatin-1 and liproxstatin-1 and the role of lipid peroxidation in ferroptotic cell death. ACS Central Science.

[46] Lim J.C., Donaldson P.J. (2011). Focus on molecules: the cystine/glutamate exchanger (system xc-). Experimental Eye Research.

[47] Conrad M., Sato H. (2012). The oxidative stress-inducible cystine/glutamate antiporter, system x c -: cystine supplier and beyond. Amino Acids.

[48] Koppula P., Zhang Y., Zhuang L., Gan B. (2018). Amino acid transporter SLC7A11/xCT at the crossroads of regulating redox homeostasis and nutrient dependency of cancer. Cancer Communications.

[49] Sato M., Kusumi R., Hamashima S., Kobayashi S., Sasaki S., Komiyama Y., Izumikawa T., Conrad M., Bannai S., Sato H. (2018). The ferroptosis inducer erastin irreversibly inhibits system xc- and synergizes with cisplatin to increase cisplatin's cytotoxicity in cancer cells. Scientific Reports.

[50] Ji X., Qian J., Rahman S.M.J., Siska P.J., Zou Y., Harris B.K., Hoeksema M.D., Trenary I.A., Heidi C., Eisenberg R., Rathmell J.C., Young J.D., Massion P.P. (2018). xCT (SLC7A11)-mediated metabolic reprogramming promotes non-small cell lung cancer progression. Oncogene.

[51] Yang Y., Yee D. (2014). IGF-I regulates redox status in breast cancer cells by activating the amino acid transport molecule xC. Cancer Research.

[52] Timmerman L., Holton T., Yuneva M., Louie R., Padró M., Daemen A., Hu M., Chan D., Ethier S., van 't Veer L., Polyak K., McCormick F., Gray J. (2013). Glutamine sensitivity analysis identifies the xCT antiporter as a common triple-negative breast tumor therapeutic target. Cancer Cell.

[53] Guo W., Zhao Y., Zhang Z., Tan N., Zhao F., Ge C., Liang L., Jia D., Chen T., Yao M., Li J., He X. (2011). Disruption of xCT inhibits cell growth via the ROS/autophagy pathway in hepatocellular carcinoma. Cancer Letters.

[54] Tsuchihashi K., Okazaki S., Ohmura M., Ishikawa M., Sampetrean O., Onishi N., Wakimoto H., Yoshikawa M., Seishima R., Iwasaki Y., Morikawa T., Abe S., Takao A., Shimizu M., Masuko T., Nagane M., Furnari F.B., Akiyama T., Suematsu M., Baba E., Akashi K., Saya H., Nagano O. (2016). The EGF receptor promotes the malignant potential of glioma by regulating amino acid transport system xc(—). Cancer Research.

[55] Ishimoto T., Nagano O., Yae T., Tamada M., Motohara T., Oshima H., Oshima M., Ikeda T., Asaba R., Yagi H., Masuko T., Shimizu T., Ishikawa T., Kai K., Takahashi E., Imamura Y., Baba Y., Ohmura M., Suematsu M., Baba H., Saya H. (2011). CD44 variant regulates redox status in cancer cells by stabilizing the xCT subunit of system xc(-) and thereby promotes tumor growth. Cancer Cell.

[56] Jiang L., Kon N., Li T., Wang S.-J., Su T., Hibshoosh H., Baer R., Gu W. (2015). Ferroptosis as a p53-mediated activity during tumour suppression. Nature.

[57] Zhang Y., Shi J., Liu X., Feng L., Gong Z., Koppula P., Sirohi K., Li X., Wei Y., Lee H., Zhuang L., Chen G., Xiao Z.-D., Hung M.-C., Chen J., Huang P., Li W., Gan B. (2018). BAP1 links metabolic regulation of ferroptosis to tumour suppression. Nature Cell Biology.

[58] de Vries G., Rosas-Plaza X., van Vugt M.A., Gietema J.A., de Jong S. (2020). Testicular cancer: determinants of cisplatin sensitivity and novel therapeutic opportunities. Cancer Treatment Reviews.

[59] Priyanka P.P., Yenugu S. (2021). Coiled-Coil Domain-Containing (CCDC) proteins: functional roles in general and male reproductive physiology. Reproductive Sciences.

[60] Leone V., Mansueto G., Pierantoni G.M., Tornincasa M., Merolla F., Cerrato A., Santoro M., Grieco M., Scaloni A., Celetti A., Fusco A. (2010). CCDC6 represses CREB1 activity by recruiting histone deacetylase 1 and protein phosphatase 1. Oncogene.

[61] Leone V., Langella C., Esposito F., Arra C., Palma G., Rea D., Paciello O., Merolla F., De Biase D., Papparella S., Celetti A., Fusco A. (2015). Ccdc6 knock-in mice develop thyroid hyperplasia associated to an enhanced CREB1 activity. Oncotarget.

[62] Luise C., Merolla F., Leone V., Paladino S., Sarnataro D., Fusco A., Celetti A. (2012). Identification of sumoylation sites in CCDC6, the first identified RET partner gene in papillary thyroid carcinoma, uncovers a mode of regulating CCDC6 function on CREB1 transcriptional activity. PloS One.

[63] Chieffi P., De Martino M., Esposito F. (2019). New anti-cancer strategies in testicular germ cell tumors, recent patents on anti-cancer drug. Discovery.

