# Supplementary material for: The tumour suppressor CCDC6 is involved in ROS tolerance and neoplastic transformation by evading ferroptosis
Source: Heliyon. 2021 Nov 15;7(11):e08399. doi: 10.1016/j.heliyon.2021.e08399 (PMC8605351; doi:10.1016/j.heliyon.2021.e08399)

Figure 1E

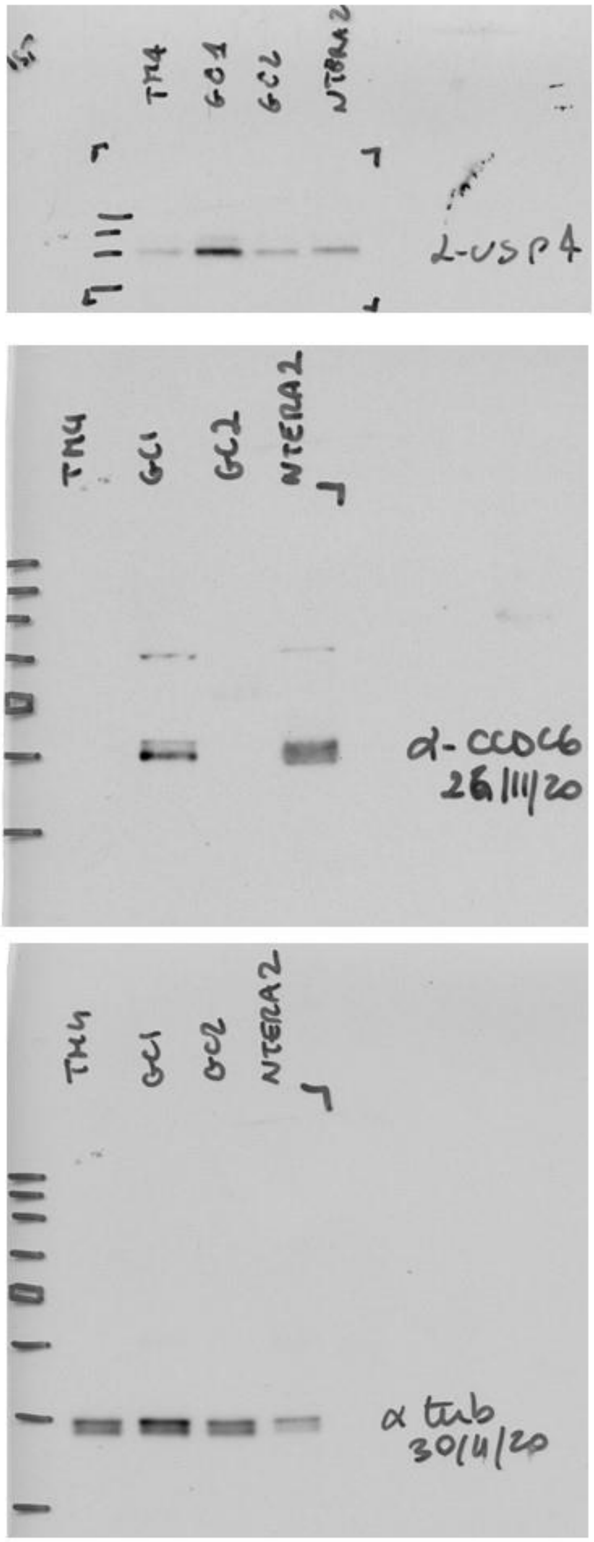
TM4
GC1
GC2
NTERA2
α-USP4
TM4
GC1
GC2
NTERA2
α-CCDC6
26/11/20
TM4
GC1
GC2
NTERA2
α tub
30/11/20

Figure 2A

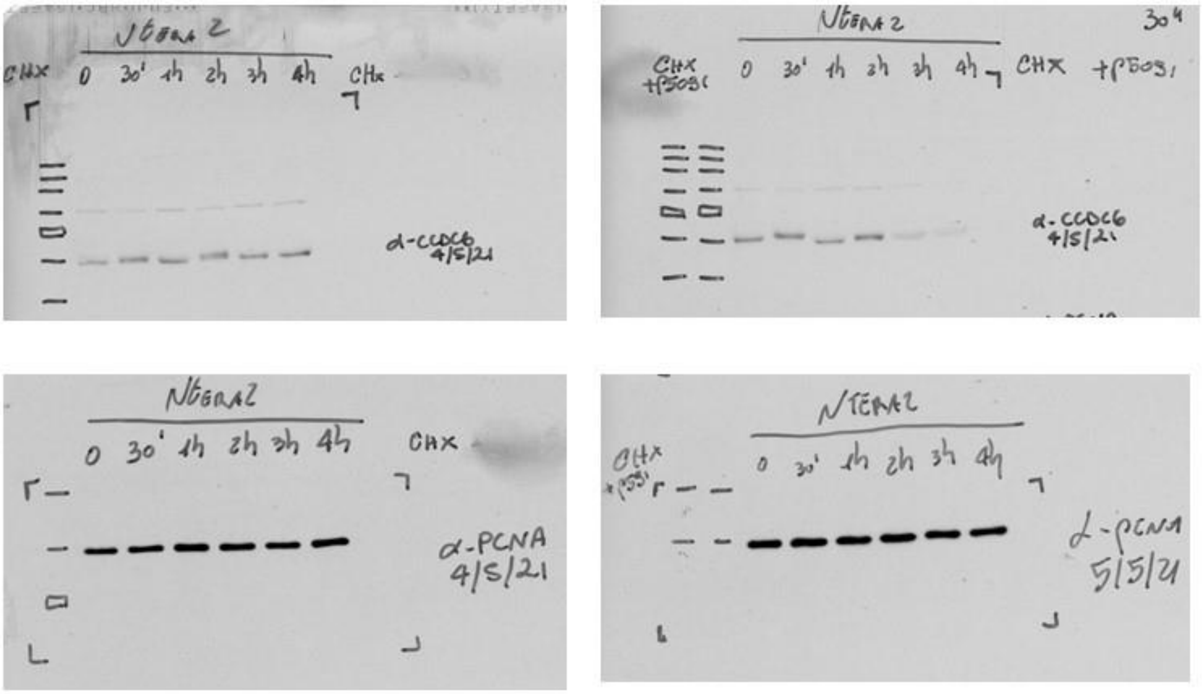

Figure 2B

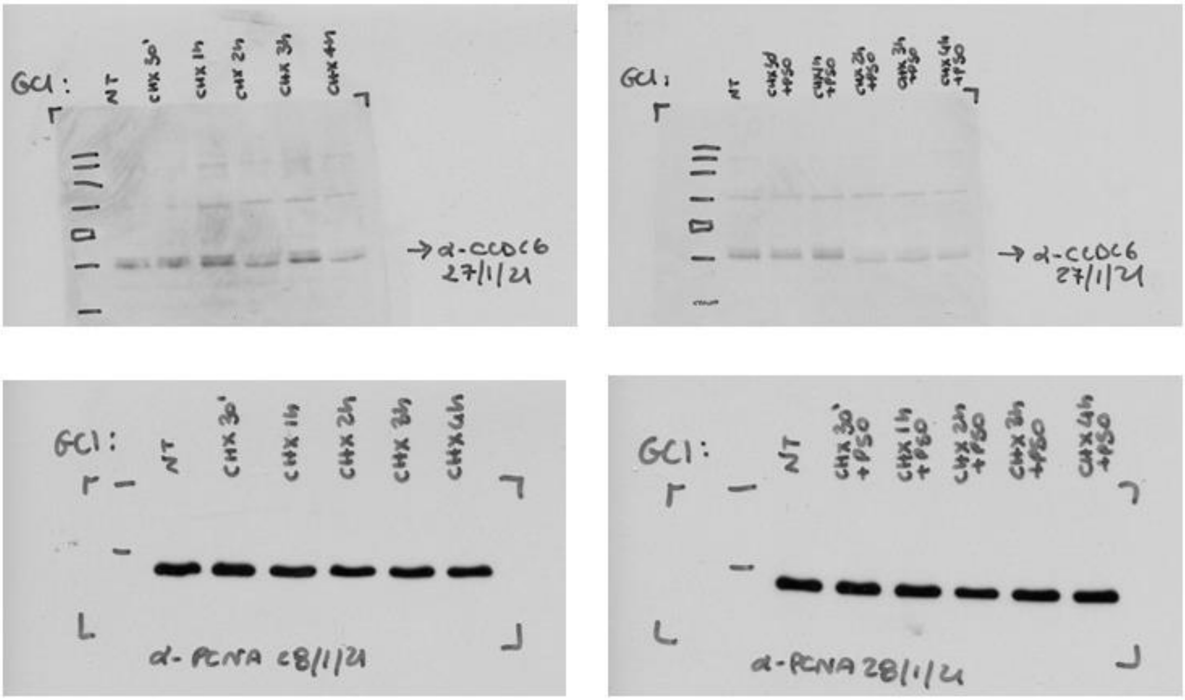

Figure 2C

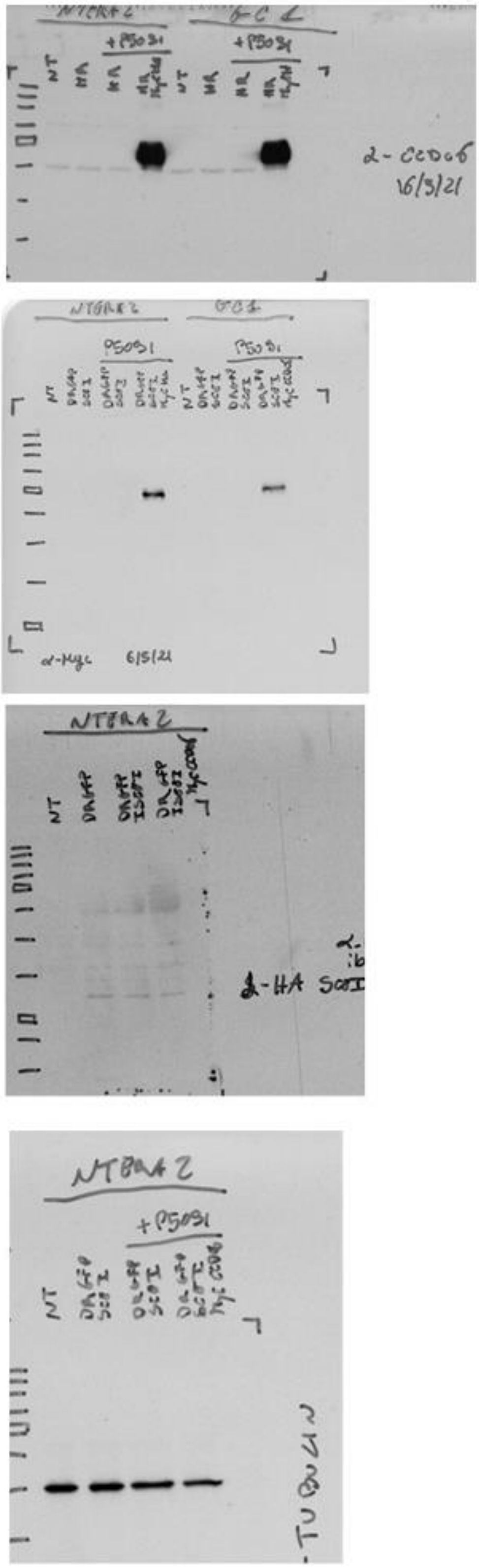

Figure 2D

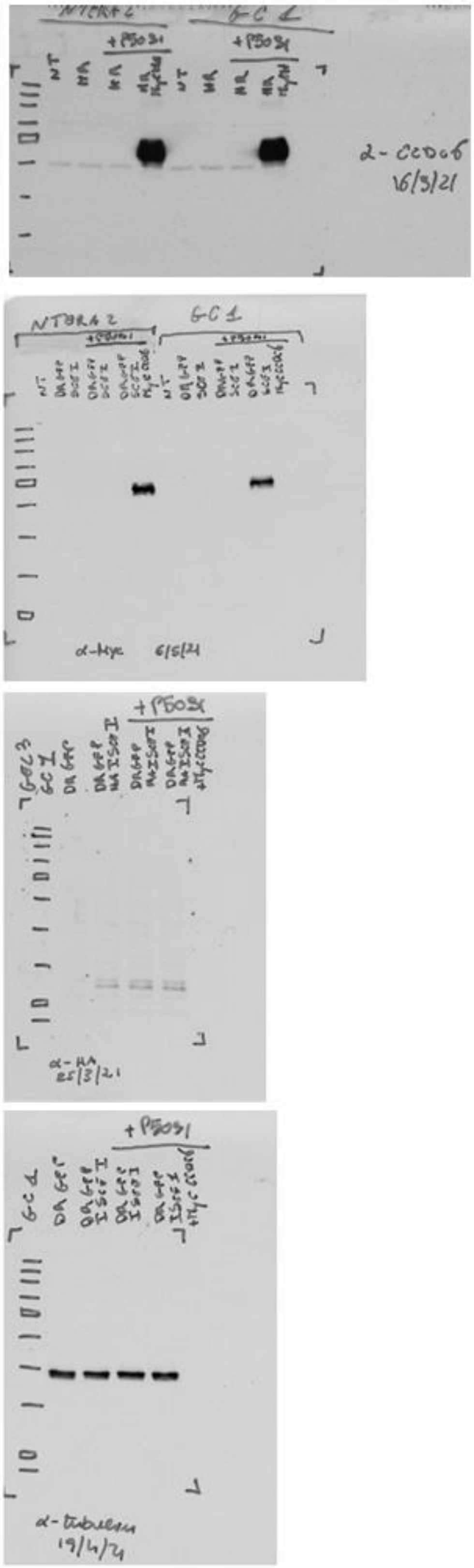

Figure 2E

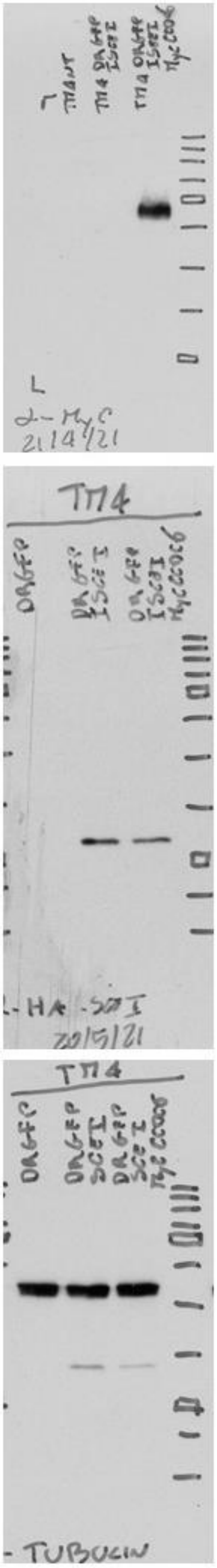

Figure 2F

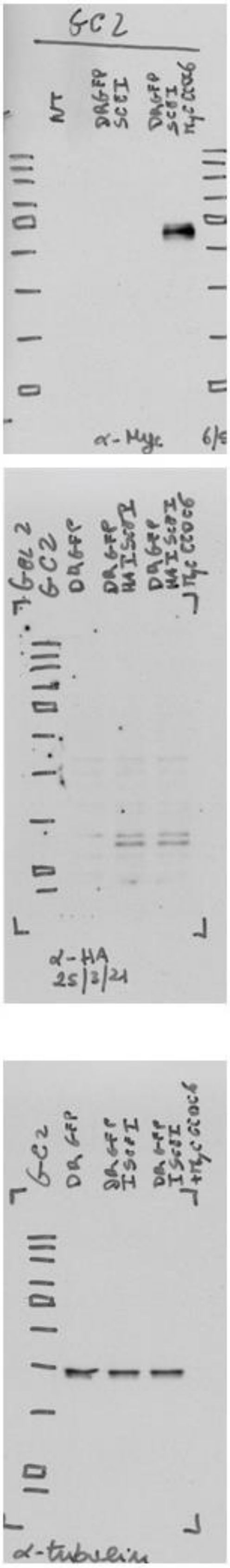

Figure 3B

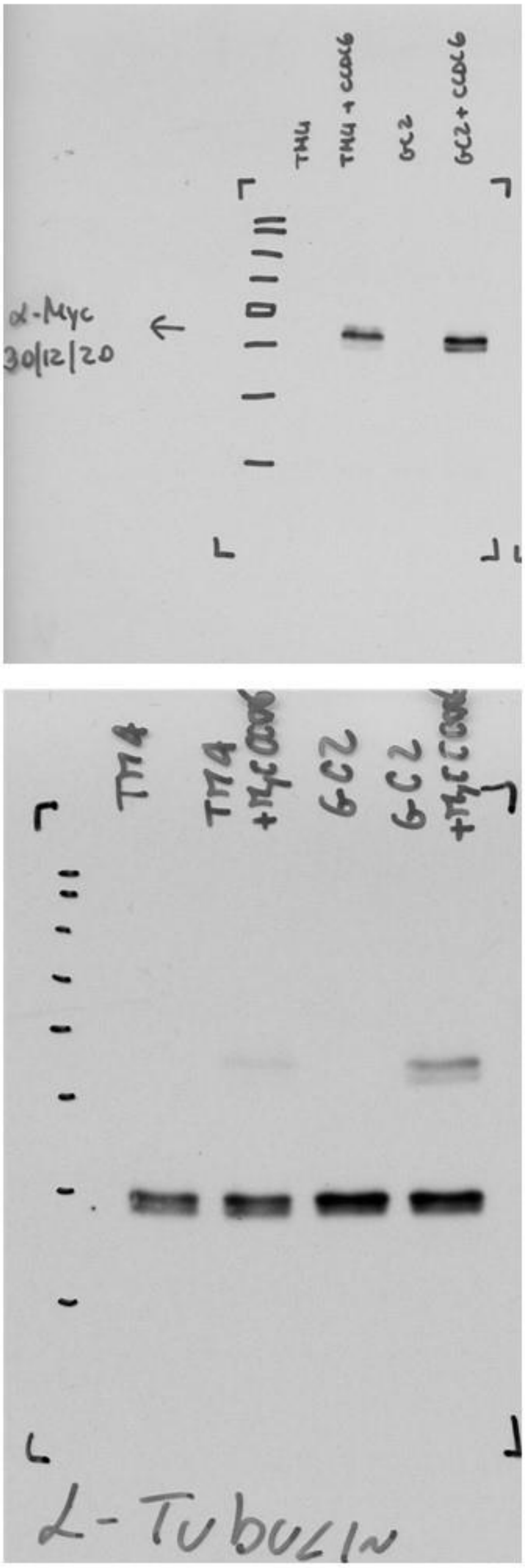
α-Myc
30/12/20
TM4
TM4 + CCDC6
GC2
GC2 + CCDC6
TM4
TM4 +Myc CCDC6
GC2
GC2 +Myc CCDC6
α-Tubulin

Figure 4A

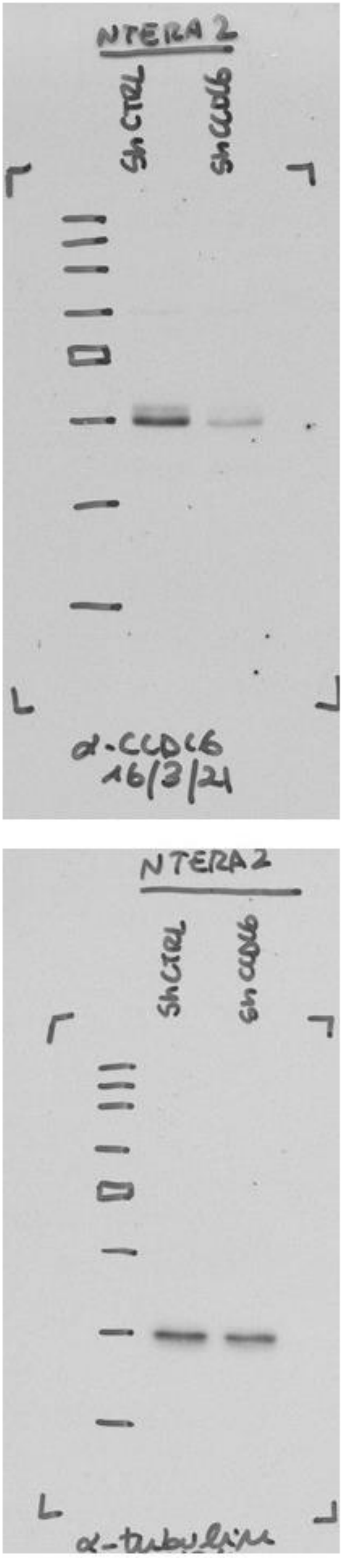

Figure 4B

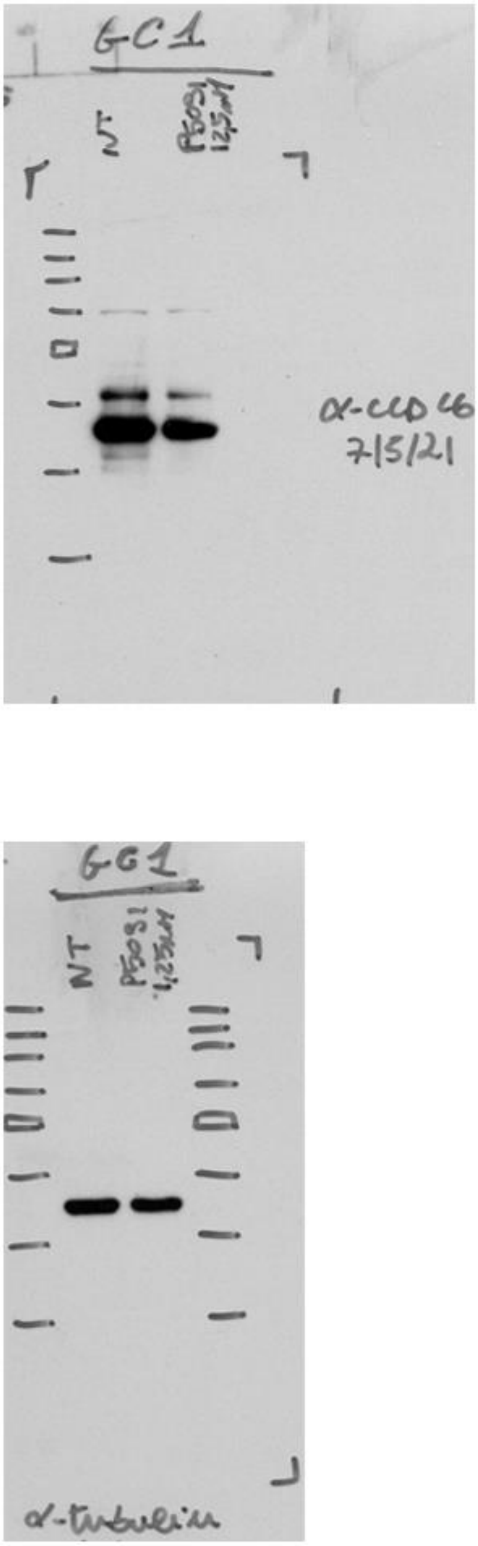

Figure 4C

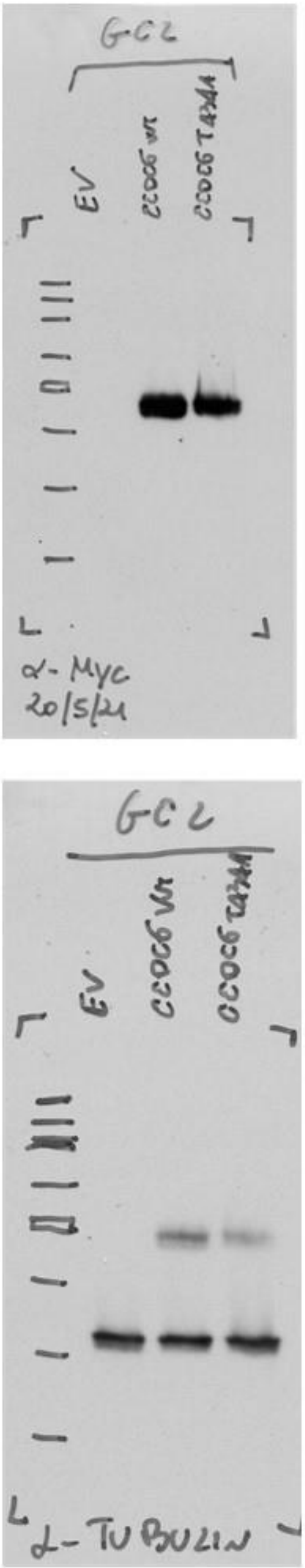

Figure 4D

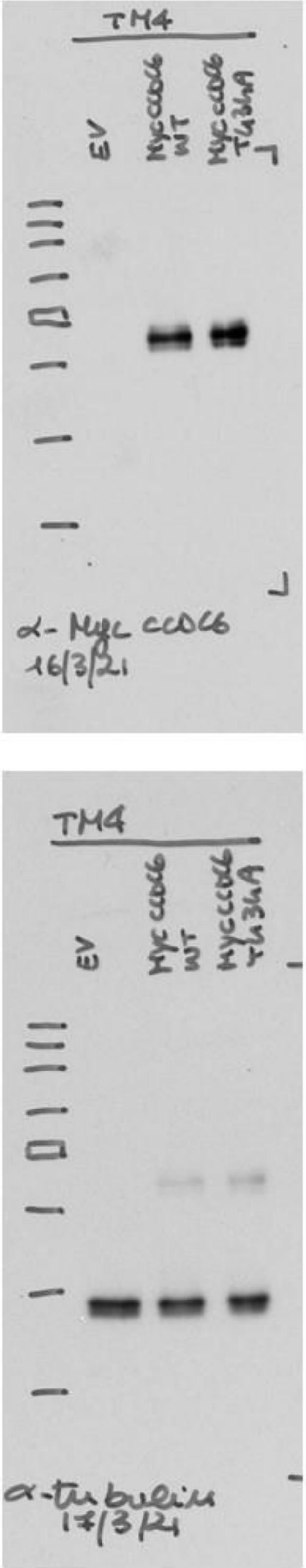

Figure 5E

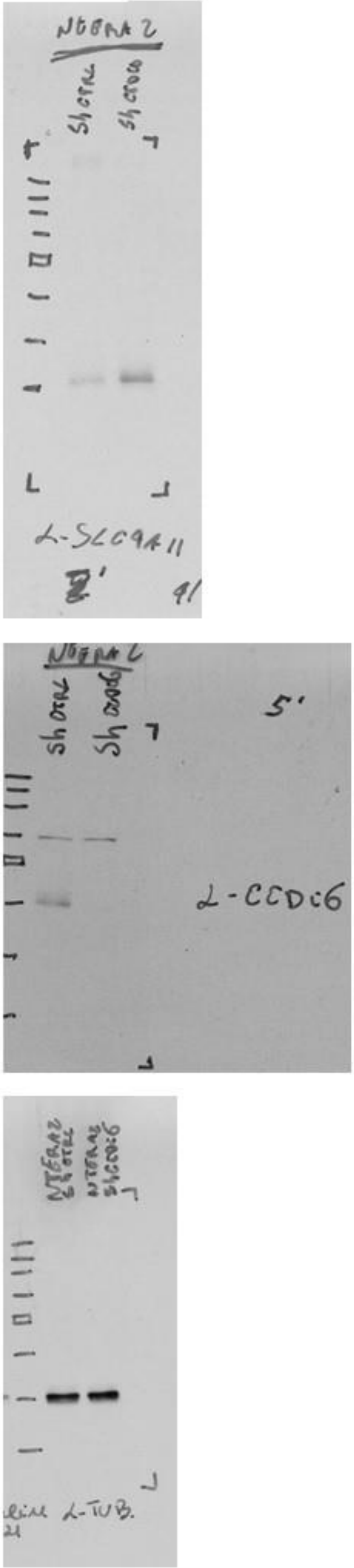

Figure 5F

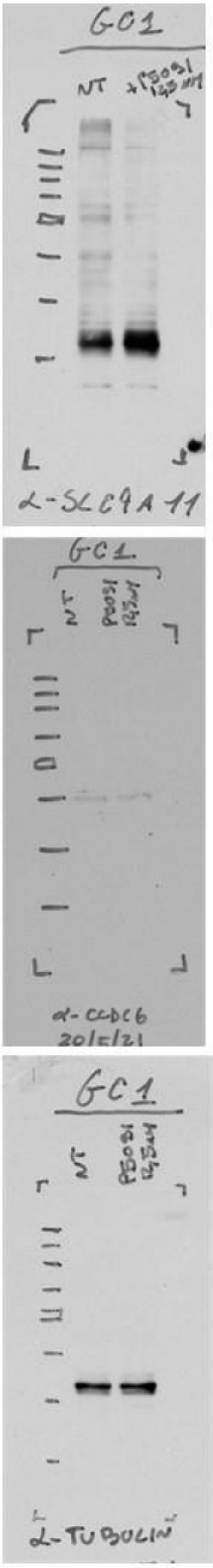

Figure 5G

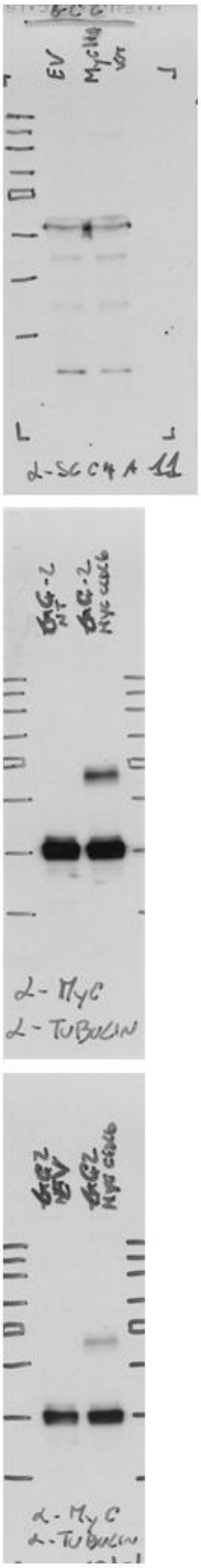

Figure 5H

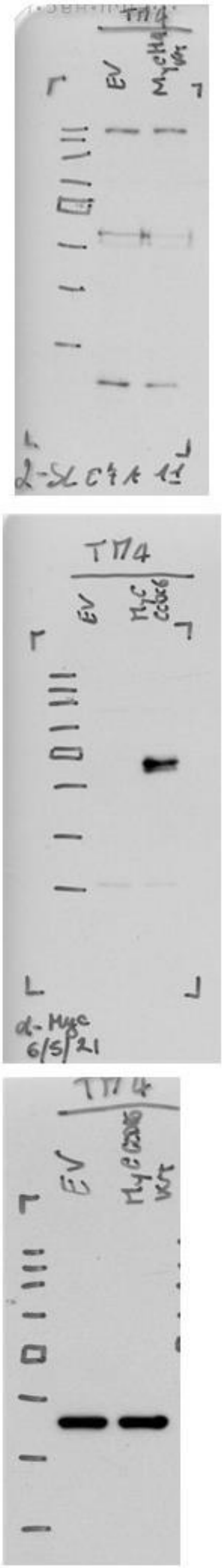

Figure 7

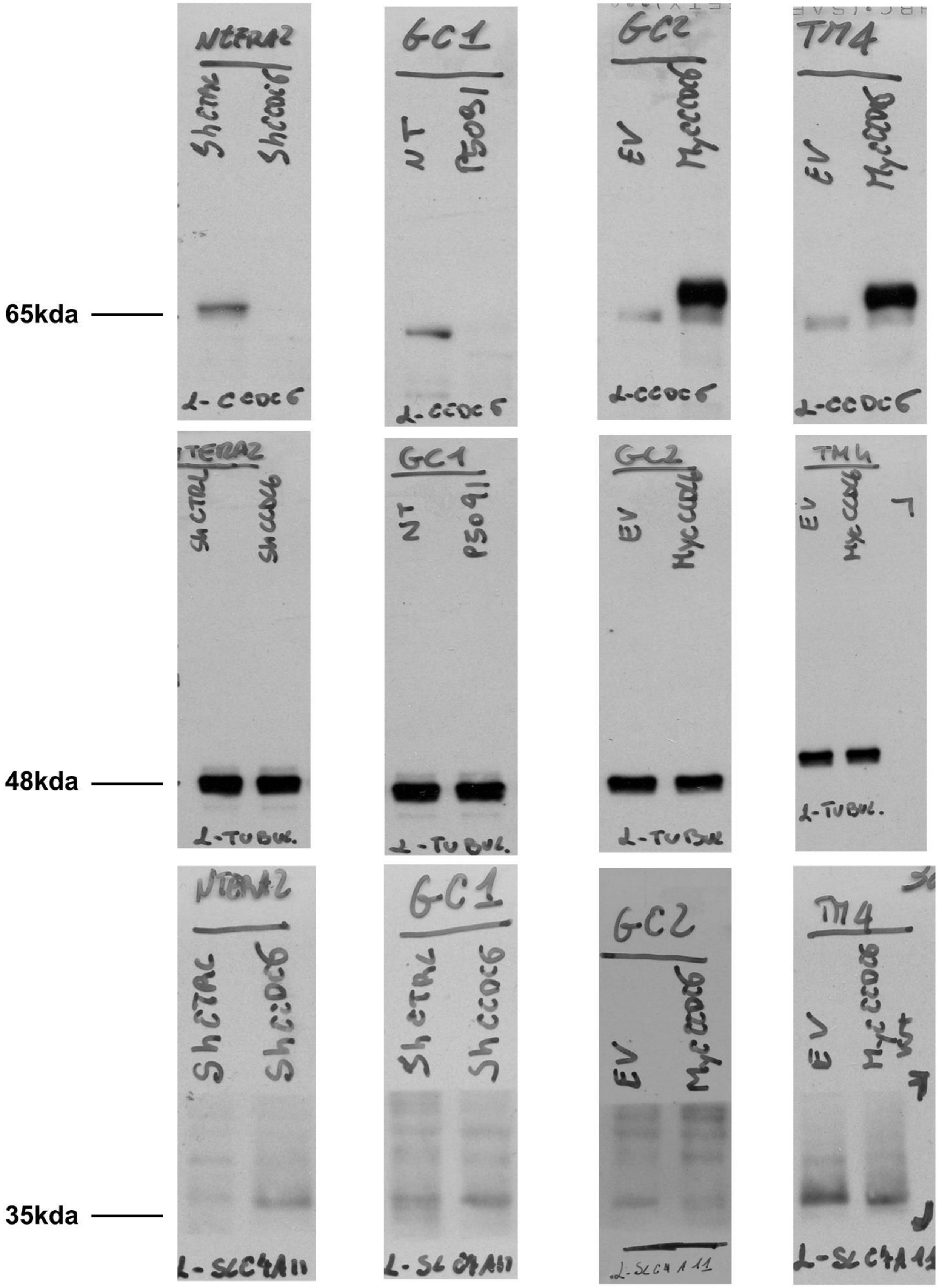

Supplementary Figure 1B

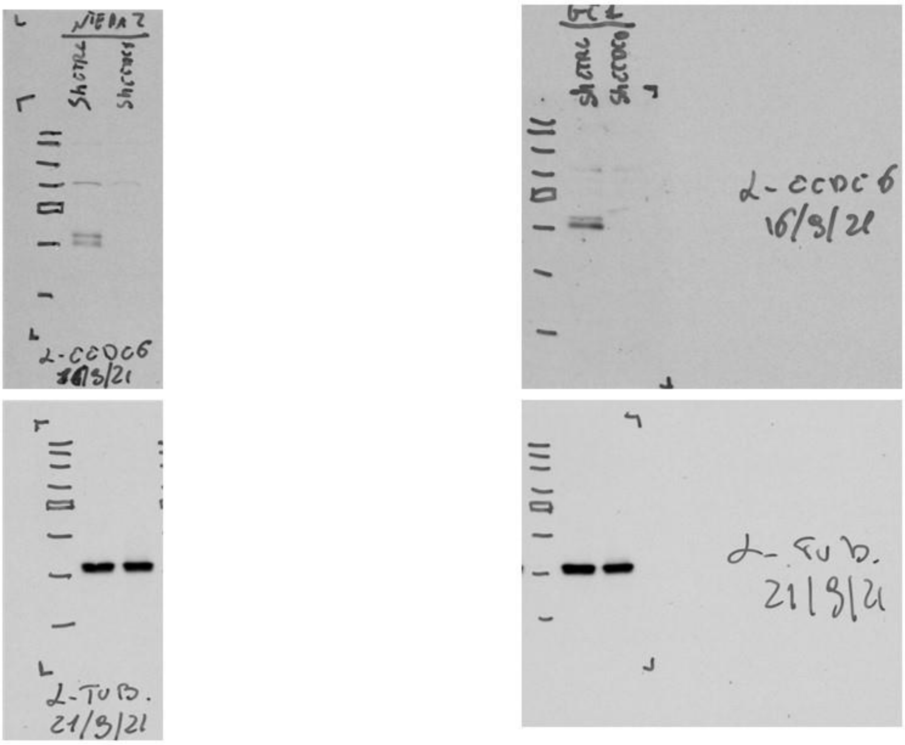

Supplementary Figure 2

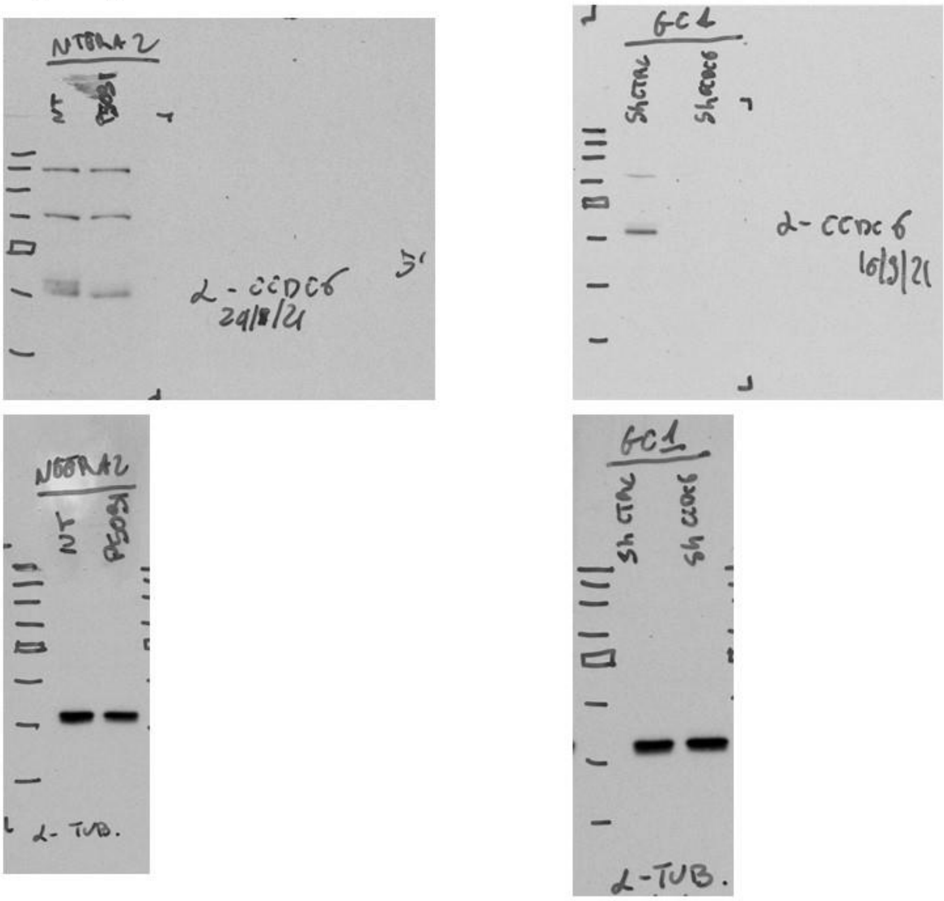

Supplement: Supplementary Figure 4.pdf — The uncropped versions of Figure 1E, Figure 2, Figure 3B, Figure 4, Figure 5, Figure 7, Supplementary Figure 1B and Supplementary Figure 2 are shown. [file mmc4.pdf]
